# Inoculation with rumen fluid in early life accelerates the rumen microbial development and favours the weaning process in goats

**DOI:** 10.1186/s42523-021-00073-9

**Published:** 2021-01-19

**Authors:** Juan Manuel Palma-Hidalgo, Elisabeth Jiménez, Milka Popova, Diego Pablo Morgavi, Antonio Ignacio Martín-García, David Rafael Yáñez-Ruiz, Alejandro Belanche

**Affiliations:** 1 Estación Experimental del Zaidín (CSIC), Profesor Albareda 1, 18008 Granada, Spain; 2 Université Clermont Auvergne, INRAE, VetAgro Sup, UMR Herbivores, F-63122 Saint-Genès Champanelle, France

**Keywords:** Core microbial community, Rumen fluid inoculation, Rumen microbial colonization, Weaning

## Abstract

**Background:**

Newborn ruminants possess an underdeveloped rumen which is colonized by microorganisms acquired from adult animals and the surrounding environment. This microbial transfer can be limited in dairy systems in which newborns are separated from their dams at birth. This study explores whether the direct inoculation of fresh or autoclaved rumen fluid from adult goats to newborn kids has a beneficial effect on rumen microbial development and function.

**Results:**

Repetitive inoculation of young kids with fresh rumen fluid from adult goats adapted to forage (RFF) or concentrate diets (RFC) accelerated microbial colonization of the rumen during the pre-weaning period leading to high protozoal numbers, a greater diversity of bacterial (+ 234 OTUs), methanogens (+ 6 OTUs) and protozoal communities (+ 25 OTUs) than observed in control kids (CTL) without inoculation. This inoculation also increased the size of the core bacterial and methanogens community and the abundance of key rumen bacteria (*Ruminococcaceae, Fibrobacteres, Veillonellaceae, Rikenellaceae, Tenericutes*), methanogens (*Methanobrevibacter ruminantium*, *Methanomicrobium mobile* and *Group 9*), anaerobic fungi (*Piromyces* and *Orpinomyces*) and protozoal taxa (*Enoploplastron, Diplodinium, Polyplastron, Ophryoscolex, Isotricha* and *Dasytricha*) before weaning whereas CTL kids remained protozoa-free through the study. Most of these taxa were positively correlated with indicators of the rumen microbiological and physiological development (higher forage and concentrate intakes and animal growth during the post-weaning period) favoring the weaning process in RFF and RFC kids in comparison to CTL kids. Some of these microbiological differences tended to decrease during the post-weaning period, although RFF and RFC kids retained a more complex and matured rumen microbial ecosystem than CTL kids. Inoculation with autoclaved rumen fluid promoted lower development of the bacterial and protozoal communities during the pre-weaning period than using fresh inocula, but it favored a more rapid microbial development during the post-weaning than observed for CTL kids.

**Conclusions:**

This study demonstrated that inoculation of young ruminants with fresh rumen fluid from adult animals accelerated the rumen microbial colonization which was associated with an earlier rumen functional development. This strategy facilitated a smoother transition from milk to solid feed favoring the animal performance during post-weaning and minimizing stress.

**Supplementary Information:**

The online version contains supplementary material available at 10.1186/s42523-021-00073-9.

## Background

Ruminants possess a complex gastric system composed of four chambers of which the rumen is the largest and hosts a vast and diverse microbial community composed of bacteria, methanogenic archaea, protozoa and fungal species which, are adapted to thrive in anaerobic conditions and are responsible for the fermentation of the diet consumed by the animal. At birth, however, the rumen is not fully developed (proto-rumen) and lacks the microbiota present in adult animals [1, 2]. Newborn ruminants rely on milk-based diets that bypass the rumen through the esophageal groove to reach the abomasum where digestion starts [3]. A correct transition from proto-rumen to rumen will later determine the efficiency of the nutrients’ digestion and absorption in the gut and other tissues [4, 5]. In this transition, microbial colonization occurring during and after birth plays a pivotal role on the development of the rumen that undergoes dramatic changes through the first weeks of life up to weaning and beyond [1, 6]. Previous studies have shown that early colonization events shape the composition of the rumen microbiome throughout life [7, 8]. The early colonizing microbes may facilitate the establishment of functional gut microbiota by several possible mechanisms. The first colonizers are facultative anaerobes and are thought to render the gut environment suitable to anaerobic rumen microbes [9]. Recent works have reported the unique and potentially important influences of maternal microbiota from the skin, udder, vagina, saliva and colostrum, which each appears to make early contributions to the bio-spatial and longitudinal succession of microbes throughout the early life of the animal [10, 11]. This is particularly critical in the context of modern dairy livestock systems in which the newborn is taken away from the mother after birth, generally fed on artificial milk, and are kept isolated from adult animals, which can limit the rumen microbial development and animal performance [5]. The magnitude of the detrimental effects increases when artificial rearing is combined with early weaning programs to minimize milk-replacer costs, which may lead to weaning-associated health and digestive problems [12].

We have previously shown that the natural rearing of newborns with the dam accelerates the rumen microbial colonization as compared to artificial milk feeding animals [1, 13], having positive effects on feed digestion and animal growth later in life [5]. This early established microbiota seems to facilitate an earlier acquisition of the digestive capacity to ferment solid feed and fiber thanks to a more diverse prokaryotic community, and also to the presence of rumen protozoa that cannot colonize the rumen unless there is direct contact between young and adult animals. Different studies have investigated the potential of inoculation young ruminants with rumen fluid from adult animals to overcome the deficient colonization process occurring under artificial milk feeding with contrasting results [14–16]. However, they have used an intermittent inoculation approach, and the analyses are limited to the most commonly studied bacteria but not to methanogens and eukaryotes (protozoa and fungi) that, despite contributing up to 50% of the total microbial biomass, are usually neglected in rumen microbiome studies [17]. The large variability observed in past studies using rumen fluid inoculation suggests that more attention must be given to the selection of the microbial inoculum and the time window in which the inoculation is applied. From an ecological perspective, the simpler and less diverse gastro-intestinal microbiota of newborn kids is more receptive to exogenous inoculation than in adult animals because it has less colonization resistance [7]. This suggests that divergent rumen microbiotas adapted to different diets could be potentially inoculated into young ruminants to modulate the colonization pattern and the establishment of a desirable rumen microbial activity for a particular production system.

In previous works, we optimized the type of inocula [18] and we showed [19] that early-in-life inoculation of goat kids with rumen fluid from adult animals stimulated feed intake and rumen function in terms of volatile fatty acids (VFA) production during the pre-weaning period. This inoculation also helped the transition from liquid to solid feeding and to minimize the growth retardation following weaning. However, the impact of the inoculation on the rumen microbiome and the identity of the key microbes which promote these physiological advantages are yet unknown. Here, we hypothesize that an early-in-life rumen microbial inoculation would modify its microbial colonization and development processes with potential long-lasting effects in goats. We used a multi-kingdom meta-taxonomic community analysis (including bacteria, methanogens, protozoa and anaerobic fungi) to get a detailed description of the rumen microbiome and how it is modulated by inocula adapted to different diets. We studied the effect of forage- and concentrate-adapted inocula as well as autoclaved rumen fluid to investigate this hypothesis.

## Results

### Inocula characterization and effects on rumen fermentation and animal performance

This study investigated the effects of early-in-life inoculation of newborns goat kids with rumen fluid from adult goats adapted to forage (RFF) or concentrate-rich diets (RFC), autoclaved rumen fluid (AUT) or absence of inoculation as control (CTL). Results indicated that RFF inocula had greater pH and acetate molar proportion, whereas RFC had greater DM content, total VFA concentration and propionate and butyrate molar proportions (Suppl. Table S1). The study of the microbial taxonomy composition also showed differences across inocula: RFF inocula had higher bacterial and methanogens OTUs richness and higher abundances of certain taxa such as *Clostridiales*, *Rikenellaeae*, *Methanomassiliicoccaceae*, *Dasytricha* and *Caecomyces*. On the contrary, RFC inocula had a higher concentration of total bacteria, protozoa and anaerobic fungi, as well as higher abundances of *Lachnospiraceae*, *Veillonellaceae*, *Methanobrevibacter*, *Polyplastron* and *Piromyces* (Suppl. Table S1). AUT inocula had a concentration of fermentation products in between RFF and RFC values, but without viable cells, as noted by the undetectable concentration of microbial DNA and intact protozoal cells after optical inspection.

**Fig. 1:**
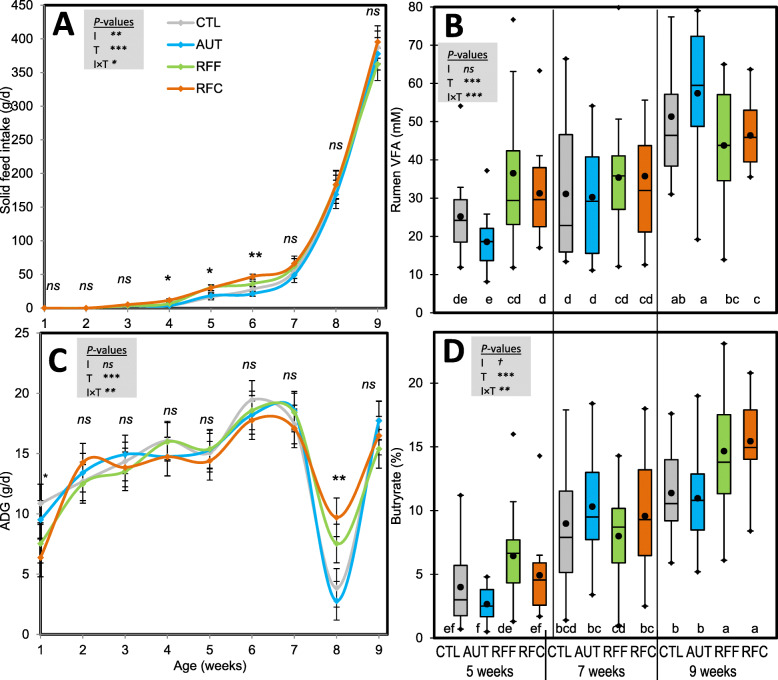
Effect of early-in-life rumen microbial inoculation on animal performance in goats. A) feed intake, B), rumen total VFA, C) animal growth and D) rumen butyrate molar proportion. Goat kids were inoculated (I) with fresh rumen fluid from adult goats adapted to forage-rich (RFF) or concentrate-rich diets (RFC), autoclaved rumen fluid (AUT) or absence of inoculation as control (CTL) and sampled at different times (T). In Figs. B and D, treatment means with different letters differ

Goat kids were daily inoculated from birth to 11 weeks of age and reared under artificial milk feeding. Rumen samples were taken at 5, 7 and 9 weeks of age to describe the rumen microbiome before, during and after weaning, respectively. Inoculation with fresh rumen (RFF and RFC) fluid increased solid feed intake (Fig. 1a) and the rumen VFA concentration during the pre-weaning period (Fig. 1b). This inoculation favored the transition to a solid diet as greater average daily gain (ADG) and butyrate molar proportion were observed during the post-weaning period (week 8, Fig. 1c) in comparison with CTL and AUT kids.

### Effect of inoculation on the multi-kingdom rumen community

The multi-kingdom analysis included all microbial OTUs from bacterial (88%), methanogens (2.7%), protozoal (2.2%) and anaerobic fungal origin (7.1%) in a combined community representing the entire rumen microbiome. Permutational analysis of variance (PERMANOVA, Table 1) showed that this multi-kingdom community was significantly affected (*P* < 0.01) by the microbial inoculation (explaining 21.8% of the total variance), the age of the animals (16.2%) and their interaction (9.9%). Pair-wise analysis also showed differences between all four inoculation treatments and sampling times being illustrated in the Principal Coordinate Analysis (PCoA, Suppl. Fig. S1). This graphical representation showed that PCO1 discriminated between control (right), AUT (center) and RFF and RFC (left) whereas the PCO2 discriminated between pre-weaning (top) and post-weaning (bottom). Samples from RFF and RFC animals positively correlated with OTUs belonging to *Ruminococcaceae, Christensenellaceae*, *Clostridiales, Anaerovorax, Mogibacterium, Bacteroidales and Prevotella* at 5 weeks, with *Entodinium* at 7 weeks and with *Prevotellaceae*, *Selenomonas*, *Lachnospiraceae* and *Succinivibrio* at 9 weeks of age, indicating a successional colonization process (Suppl. Fig. S1). For a more detailed description of the rumen microbiome, the main microbial groups were analyzed separately.

**Table 1:** Permutational analysis of variance describing the effects of early-in-life rumen microbial inoculation and time on the rumen community structure

Community^1^	Inoculation	Time	Interaction
Multi-kingdom			
Variance (%)	21.8	16.2	9.90
Pseudo-F	12.3	13.7	2.79
*P*-value	< 0.001	< 0.001	< 0.001
Bacteria			
Variance (%)	16.1	17.3	9.26
Pseudo-F	8.20	13.21	2.36
*P*-value	< 0.001	< 0.001	< 0.001
Methanogens			
Variance (%)	24.9	11.5	9.07
Pseudo-F	12.5	8.70	2.28
*P*-value	< 0.001	< 0.001	0.002
Protozoa			
Variance (%)	14.5	9.07	6.28
Pseudo-F	6.79	4.23	1.47
*P*-value	< 0.001	< 0.001	0.096
Anaerobic fungi			
Variance (%)	30.4	1.73	4.41
Pseudo-F	8.45	1.44	1.23
*P*-value	< 0.001	0.192	0.193

^1^Microbial data were log10 transformed and only Spearman’s correlations coefficients *ρ* > 0.3 (green) or *ρ* < −0.3 (red) and *p* < 0.01 are shown (*N* = 96). Parameters: milk, concentrate, forage and DM intake (g/d), rumen pH,total volatile fatty acids (mM), acetate (%), propionate, butyrate (%), odd and branched chain fatty acids (%), microbial concentration (log10 copies/mg DM), OTUs richness (−R), plasma β-hydroxybutyrate (mM), blood glucose (mg/dL), average daily gain (g/d) and ADG during the post weaning period (ADG-pw)

### Effect of inoculation on the rumen bacterial community

**Fig. 2:**
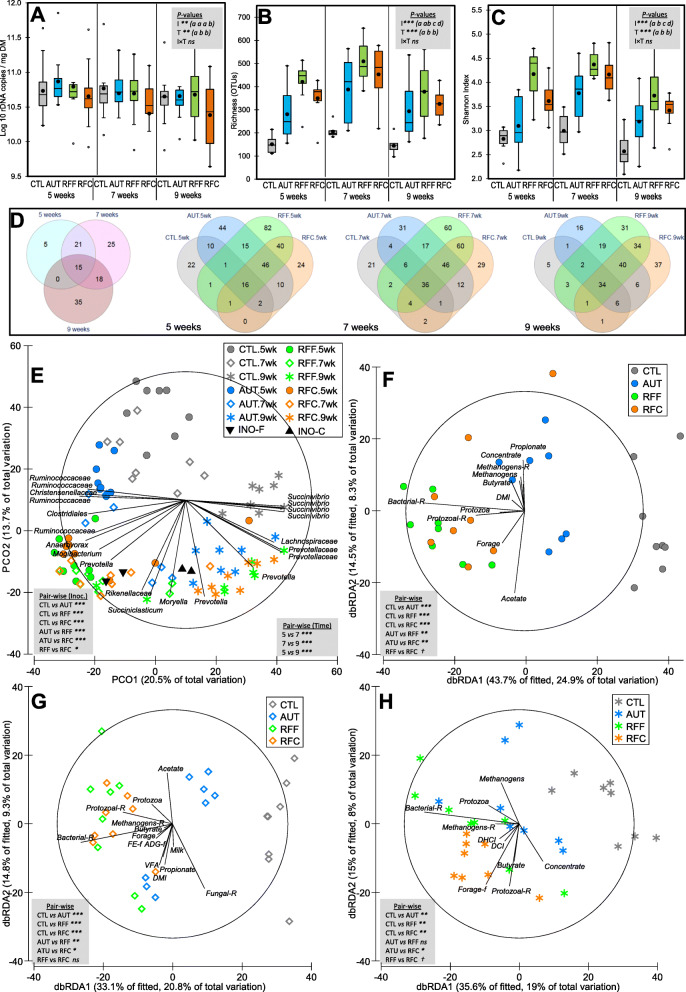
Effects of early-in-life rumen microbial inoculation on the rumen bacterial community in goats. Boxplots indicating the total bacterial abundance (**A**) and diversity indexes (**B,C**). Venn diagrams indicating the number of OTUs in the core community (**D**). Principal co-ordinates analysis (**E**) illustrating the treatment effects on the bacterial community showing the most discriminant OTUs (*ρ* > 0.75). Distance-based redundancy analysis illustrating relationship between the structure of the bacterial community and rumen function indicators before (**F**), during (**G)** and after (**H**) weaning. Pair-wise PERMANOVA values are provided in grey boxes based on the Bray-Curtis dissimilarity. Goat kids inoculated (I) with fresh rumen fluid from adult goats adapted to forage-rich (RFF) or concentrate-rich diets (RFC), autoclaved rumen fluid (AUT) or absence of inoculation as control (CTL) and sampled at different times (T). In Figs. A, B and C, treatment means with different letters differ

RFC kids had lower total bacterial abundance per gram of DM than the other three treatments (Fig. 2a). The sequencing analysis generated on average 14,451 ± 716 high quality bacterial sequences per sample. RFF kids, followed by RFC and AUT, showed the highest bacterial diversity in terms of OTUs (Fig. 2b) and Shannon index (Fig. 2c), whereas CTL kids showed the lowest bacterial diversity indexes. Venn diagrams showed that the core bacterial community was composed by 15 OTUs (Fig. 2d). The inoculation with fresh rumen promoted a large core bacterial community in RFF (composed by 202, 231 and 164 OTUs) and RFC kids (139, 190 and 159 OTUs at weeks 5, 7 and 9, respectively). Moreover, many of these OTUs (20–30%) were exclusively shared between these two treatments indicating a similar community structure. AUT kids had a medium size core community (144, 153 and 124 OTUs), whereas CTL kids had much smaller core community (53, 76 and 53 OTUs at week 5, 7 and 9, respectively), being most of these OTUs (up to 64%) common across all treatments.

PERMANOVA analysis showed that inoculation and sampling time had significant impacts on bacterial community structure, explaining 16–17% of the total variance (Table 1). PCoA illustrated these differences in the bacterial community structure (Fig. 2e) in which PCO1 captured a gradient of community development according to the age of the kids (from left to right), whereas PCO2 did so for the CTL kids (from up to down). This PCoA also identified relevant microbes which partially explained these differences. For example, samples taken at 9 weeks of age from AUT, RFF and RFC kids correlated with the presence of *Prevotella* OTUs, whereas those from CTL kids did so with *Succinivibrio* OTUs. Inocula samples clustered together with samples from RFF and RFC kids at 7 weeks of age, indicating a similar bacterial community. The effect of the inoculation differed with the age of the kids (interaction, *P* < 0.001. Table 1), indicating that specific analyses for each sampling time were needed.

During the pre-weaning period (week 5, Fig. 2f), inoculation with fresh rumen fluid showed a unique and different bacterial community as compared to AUT or CTL kids. This bacterial community correlated with indicators of the rumen microbial and functional development such as dry matter intake (DMI), forage intake, protozoal concentration and bacterial and protozoal richness. Most differences among treatments were also detected at weening (week 7, Fig. 2g). Again, clustered samples from these latter groups were related with higher bacterial and protozoal richness, as well as with the average daily gain (ADG-f) and feed efficiency (FE-f) during the following week, suggesting that this community structure minimized the weaning shock. During the post-weaning period (week 9, Fig. 2h) CTL kids retained a different bacterial community than that of other treatments, whereas the bacterial community of AUT kids became closer to those kids inoculated with fresh rumen fluid. Greater bacterial and protozoal richness were again associated with the bacterial community structure of inoculated groups, along with digestible cellulose (DCI) and hemicellulose (DHCI) and forage intake during the following week (Forage –f).

**Fig. 3:**
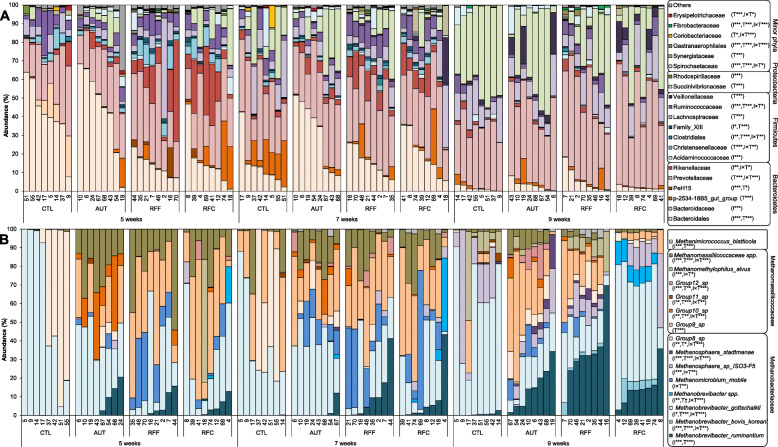
Effect of the early-in-life rumen microbial inoculation on the rumen prokaryotic taxa distribution. A) Bacterial and (B) methanogens abundances. Goat kids inoculated (I) with fresh rumen fluid from adult goats adapted to forage-rich (RFF) or concentrate-rich diets (RFC), autoclaved rumen fluid (AUT) or absence of inoculation as control (CTL) and sampled at different times (T)

The analysis of the relative abundances of the most predominant bacterial families and genera (Fig. 3a and Suppl. Table S2) indicated that 12 out of the 21 families identified showed significant differences based on the inoculation treatment, regardless of sampling time. Inoculation with fresh rumen fluid promoted the presence of a number of minority bacterial taxa at week 5 which were not present in CTL kids, the first three being also absent in AUT kids. Most of these taxa were not detected at later sampling times in CTL kids indicating a microbial colonization delay. Inoculation with fresh rumen fluid also increased the abundance of various phyla (e.g. *Firmicutes, Fibrobacteres, Tenericutes, Cyanobacteria* and *Elusimicrobia*) and genera (e.g. *Fibrobacter, Succiniclasticum, Eubacterium or Lachnoclostridium*) in comparison with CTL and AUT kids across sampling times. On the contrary, *Bacteroidales, Alloprevotella* and *Coprococcus* were most abundant in CTL and AUT groups across sampling times. The higher bacterial Shannon index observed in RFF than in RFC inocula was also noted in RFF inoculated animals, however the divergent taxa distribution observed between them was not reflected in the inoculated kids. Moreover, 17 out of the 21 taxa presented significant differences according to sampling time: *Prevotellaceae* and *Succinivibrionaceae* increased over time whereas *Ruminococcaceae* decreased. The interaction between inoculation and sampling time was significant in 10 out of 21 bacterial taxa indicating that the effects were more obvious before than after weaning.

Spearman correlations were performed to assess the potential implications of changes in rumen meta-taxonomic data on animal physiology (Table 2 and Suppl. Table S3). Bacterial richness, a number of bacterial phyla such as *Fibrobacteres*, *Firmicutes*, *Tenericutes*, *Elusimicrobia*, *Cyanobacteria*, *Chloroflexi* and *Lentisphaerae*, and several bacterial families such as *Ruminococcaceae*, *Veillonellaceae*, *Rhodocyclaceae* or *Rikenellaceae* positively correlated with various indicators of the rumen physiological development such as forage and solids intake, acetate molar proportion, presence of protozoa and higher bacterial, protozoal and methanogens diversity. Moreover, the abundance of *Firmicutes* and *Veillonellaceae* positively correlated with the ADG during the post-weaning period indicating a better transition from liquid to solid feed during the post-weaning period. On the contrary, the phylum *Bacteroidetes*, the families *Bacteroidaceae*, *Comamonadaceae* and *Neisseriaceae* showed a negative correlation with these indicators of the rumen physiological development as well as with animal performance during the post-weaning period.

**Table 2:**
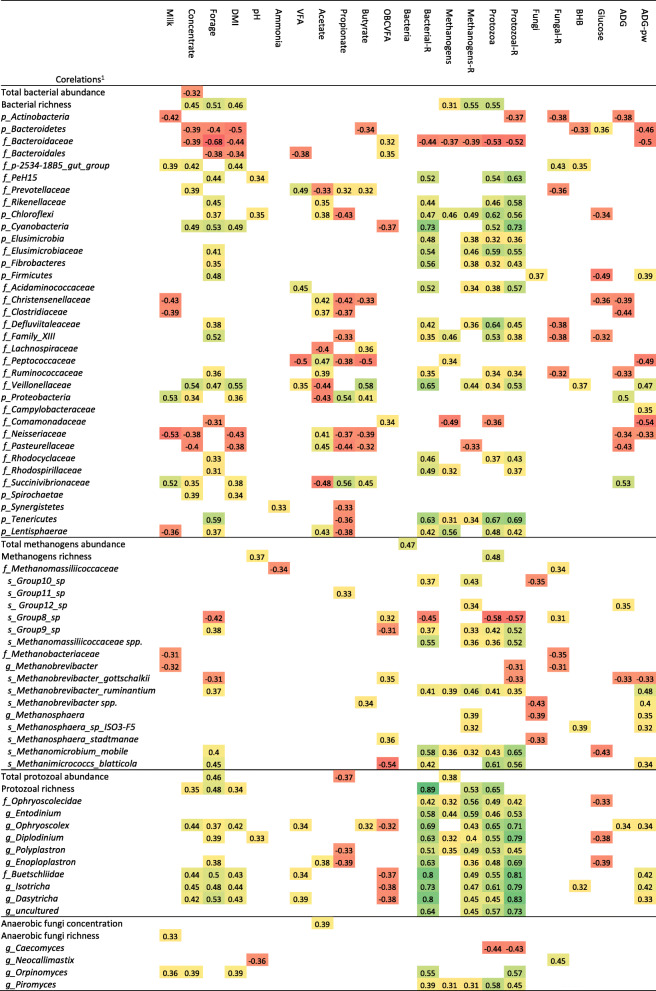
Correlations among the rumen microbiota and digestive physiology data.

### Effect of inoculation on the rumen methanogens community

**Fig. 4:**
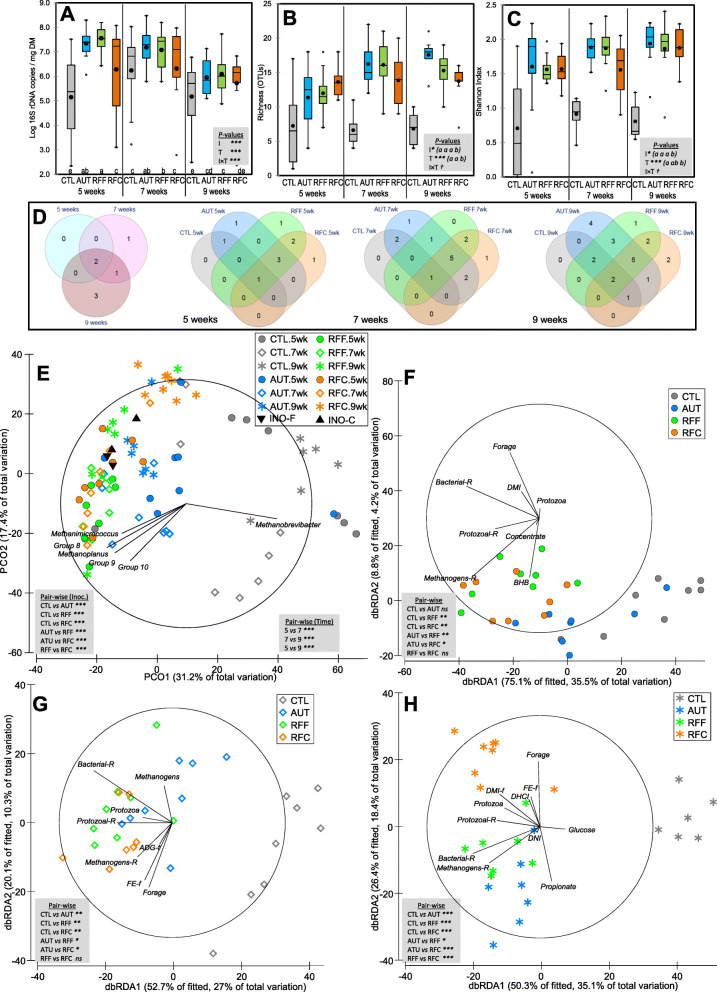
Effects of early-in-life rumen microbial inoculation on the rumen methanogens community in goats. Boxplots indicating the total methanogens abundance (**A**) and diversity indexes (**B,C**). Venn diagrams indicating the number of OTUs in the core community (**D**). Principal co-ordinates analysis (**E**) illustrating the treatment effects on the methanogens community showing the most discriminant OTUs (*ρ* > 0.55). Distance-based redundancy analysis illustrating relationship between the structure of the methanogens community and rumen function indicators before (**F**), during (**G**) and after (**H**) weaning. Pair-wise PERMANOVA values are provided in grey boxes based on the Bray-Curtis dissimilarity. Goat kids inoculated (I) with fresh rumen fluid from adult goats adapted to forage-rich (RFF) or concentrate-rich diets (RFC), autoclaved rumen fluid (AUT) or absence of inoculation as control (CTL) and sampled at different times (T). In Figs. A, B and C, treatment means with different letters differ

The interaction between inoculation treatment and time on the concentration of methanogenic archaea in the rumen was significant (Fig. 4a). Although RFF kids, followed by AUT and RFC, showed the highest concentrations of methanogens at 5 weeks of age (and CTL the lowest) these differences tended to decrease as kids aged. Sequencing analysis generated an average of 14,180 ± 1100 high-quality methanogens sequences per sample and showed that methanogens diversity increased with the age of the kids (Fig. 4b). Moreover, CTL kids showed lower methanogens diversity in terms of OTUs and Shannon index (Fig. 4c) than observed in other treatments across time points. The methanogens core community was composed by only two *Methanobrevibacter* OTUs which were shared across all treatments and time points (Fig. 4d). However, at week 9 new *Methanobrevibacter* and *Methanosphaera* OTUs appeared in this core community. Venn diagrams for individual time points revealed that the methanogens core community remained similar for CTL kids (5, 3 and 5 OTUs at week 5, 7 and 9, respectively) but increased for AUT (6, 12 and 17 OTUs), RFF (7, 9 and 15 OTUs) and RFC kids (7, 11 and 13 OTUs).

Rumen methanogens community structure was greatly affected by inoculation, sampling time and their interaction (Table 1, Fig. 4e), however, PERMANOVA indicated that the proportion of the variance explained by the inoculation treatment was twice more than that explained by sampling time (24.92 vs 11.54%). PCoA showed that PCO1 separated samples from CTL (right) and from the rest of treatments (left), whereas PCO2 disaggregated samples between pre-weaning (bottom) and post-weaning (top). Moreover, samples collected from RFF and RFC at 5 weeks positively correlated with OTUs belonging to *Methanimicrococcus, Methanophanus* and Groups 8, 9 and 10.

The study of the methanogens community structure at 5, 7 and 9 weeks of age using Distance-Based Redundancy Analyses (Fig. 4f) showed a general pattern characterized by a separation through axis 1 between samples from CTL kids (right) and those from RFF and RFC kids (left). At week 5, RFF and RFC samples positively correlated with the presence of higher diversity levels for bacteria, methanogen and protozoa and higher DMI and blood beta-hydroxybutyrate indicating a more microbiological and functional rumen development. At weeks 7 (Fig. 4f) and 9 (Fig. 4h), RFF and RFC samples correlated with higher diversity indexes, protozoal concentration, forage intake and ADG and FE during the following week after weaning whereas CTL samples clustered on opposite direction indicating a more undeveloped methanogens community.

The analysis of the relative abundances of the 15 most predominant methanogen species (Fig. 3b and Suppl. Table S4) showed differences according to the inoculation treatment (13 species) and sampling time (11 species); however a significant interaction was found for most of them. At 5 weeks of age, nearly the entire methanogens community (99.7%) in CTL kids was formed by *Methanobrevibacter gottschalkii* and *Group8_sp*, however these two species only represented 39.0, 22.9 and 29.1% in AUT, RFF and RFC. On the contrary, RFF and RFF had increased abundances of *Group9_sp* (34.4%), *Methanomicrobium mobile* (9.7%), *Methanobrevibacter ruminantium* (3.8%) and Methanobassiliicoccaceae spp. (18.3%), whereas AUT kids were more abundant in *Group10_sp* (14.7%). At week 7, a consistent presence of *Methanimicrococcus blatticola*, *Methanomicrobium mobile*, *Methanosphaera*, *Group12_sp* and *Group10_sp* was detected in AUT, RFF and RFC but were absent in CTL kids which still retained higher numbers of *M. gottschalkii* (39.6%). This over-representation was even bigger at 9 weeks of age (59.9%), whereas inoculated kids were more abundant in a greater number of methanogen species (e.g. Group8_sp and Group_9sp). The differences in the methanogens community noted between RFF and RFC inocula were not observed in the inoculated kids, although RFF kids had a higher total methanogens concentration.

The abundance of *M. gottschalkii* (and *Group8_sp*) was negatively correlated with forage intake, presence of protozoa and ADG before and after weaning, indicating the presence of an immature methanogens community (Table 2). On the contrary, *M. blatticola*, *M. mobile*, *M. ruminantium*, *Methanosphaera* and *Group9_sp* were positively correlated with indicators of a greater rumen microbiological and physiological development (forage intake and ADG before and after weaning).

### Effect of inoculation on the rumen protozoal community

**Fig. 5:**
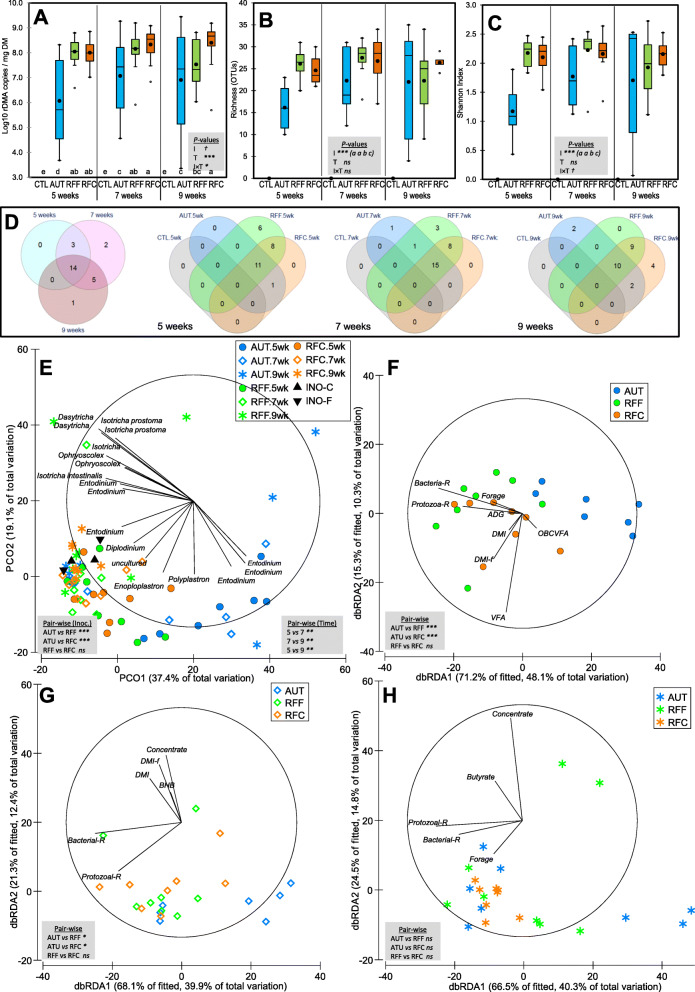
Effects of early-in-life rumen microbial inoculation on the rumen protozoal community in goats. Boxplots indicating the total protozoal abundance (**A**) and diversity indexes (**B,C**). Venn diagrams indicating the number of OTUs in the core community (**D**). Principal co-ordinates analysis (**E**) illustrating the treatment effects on the rumen protozoal community showing the most discriminant OTUs (*ρ* > 0.55). Distance-based redundancy analysis illustrating relationship between the structure of the protozoal community and rumen function indicators before (**F**), during (**G**) and after (**H**) weaning. Pair-wise PERMANOVA values are provided in grey boxes based on the Bray-Curtis dissimilarity. Goat kids inoculated (I) with fresh rumen fluid from adult goats adapted to forage-rich (RFF) or concentrate-rich diets (RFC), autoclaved rumen fluid (AUT) or absence of inoculation as control (CTL) and sampled at different times (T). In Figs. A, B and C, treatment means with different letters differ

Control kids remained protozoa-free over the entire duration of this study. Inoculation with fresh rumen fluid promoted a higher concentration of rumen protozoa (Fig. 5a) at week 5, but these differences tended to be smaller as time progressed (interaction *P* < 0.05). The 18S amplicon sequencing yielded an average of 18,164 ± 644 sequences per sample and diversity analysis showed that RFF and RFC had higher protozoal diversity in terms of OTUs (Fig. 5b) and Shannon index (Fig. 5c) than AUT kids across sampling times. A total of 14 protozoal OTUs formed the core community shared between AUT, RFF and RFC kids across time points (Fig. 5d). The treatment-specific core community increased over time for RFC (20, 23 and 25 OTUs at week 5, 7 and 9, respectively), and for RFF kids (25, 27 and 19 OTUs) since most OTUs were shared across these two treatments. On the contrary, the protozoal core community was smaller and remained constant over time for AUT kids.

PERMANOVA revealed that the inoculation and the sampling time greatly modified the protozoal community structure explaining 14.5 and 9.07% of the total variance, respectively (Table 1). Pair-wise comparisons and PCoA analysis (Fig. 5e) showed that inoculation with fresh rumen fluid promoted a protozoal community similar to the observed in the inocula and positively correlated with the presence of 8 different protozoal OTUs, whereas the community in the AUT kids only correlated with *Entodinium* OTUs. The analysis of the protozoal community at different time points showed that RFF and RFC always shared a similar protozoal community which was positively correlated with indicators of a rumen microbiological (higher bacterial and protozoal richness) and functional development (higher intakes, rumen VFA, butyrate and ADG). This protozoal community differed to that observed in AUT kids at 5 (Fig. 5f) and 7 weeks (Fig. 5g) but not at 9 weeks of age (Fig. 5h), indicating a delay in the rumen protozoal colonization in AUT kids.

**Fig. 6:**
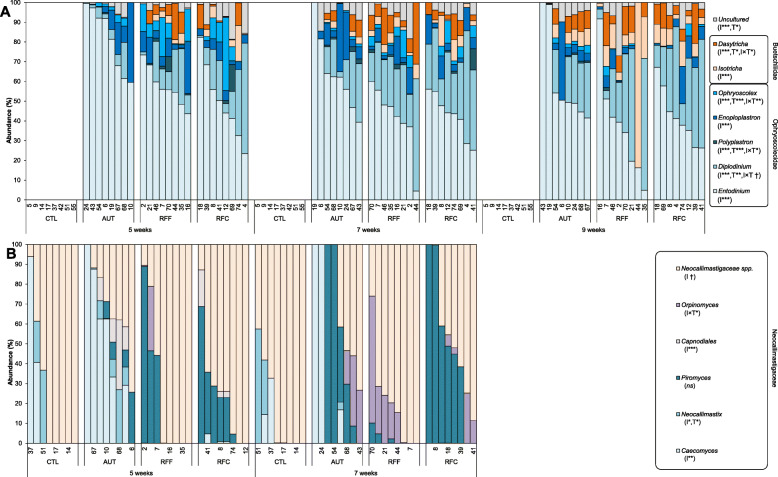
Effect of the early-in-life rumen microbial inoculation on the rumen eukaryotic taxa distribution. A) Protozoa and (B) anaerobic fungi abundances. Goat kids inoculated (I) with fresh rumen fluid from adult goats adapted to forage-rich (RFF) or concentrate-rich diets (RFC), autoclaved rumen fluid (AUT) or absence of inoculation as control (CTL) and sampled at different times (T)

Analysis of the protozoa relative abundances (Fig. 6a and Suppl. Table S5) showed a progressive decrease over time in the entodiniomorphids (family *Ophryoscolecidae*) and an increase in holotrichs protozoa (family *Buetschliidae*). AUT kids had increased numbers of *Entodinium*, whereas RFF and RFC were more abundant on *Diplodinium, Enoploplastron, Isotricha* and *Dasytricha*, these differences being greater before than after weaning. The abundance of most protozoal species was positively correlated with DM intake and bacterial, methanogens and protozoal diversities. Abundances of *Ophryoscolex*, *Isotricha* and *Dasytricha* were also correlated with higher VFA concentration and ADG during the post-weaning period as indicators of rumen development. The higher total protozoal concentration detected in RFC than in RFF inocula, was also observed in RFC kids at 9 weeks of age.

### Effect of inoculation and age on the rumen fungal community

**Fig. 7:**
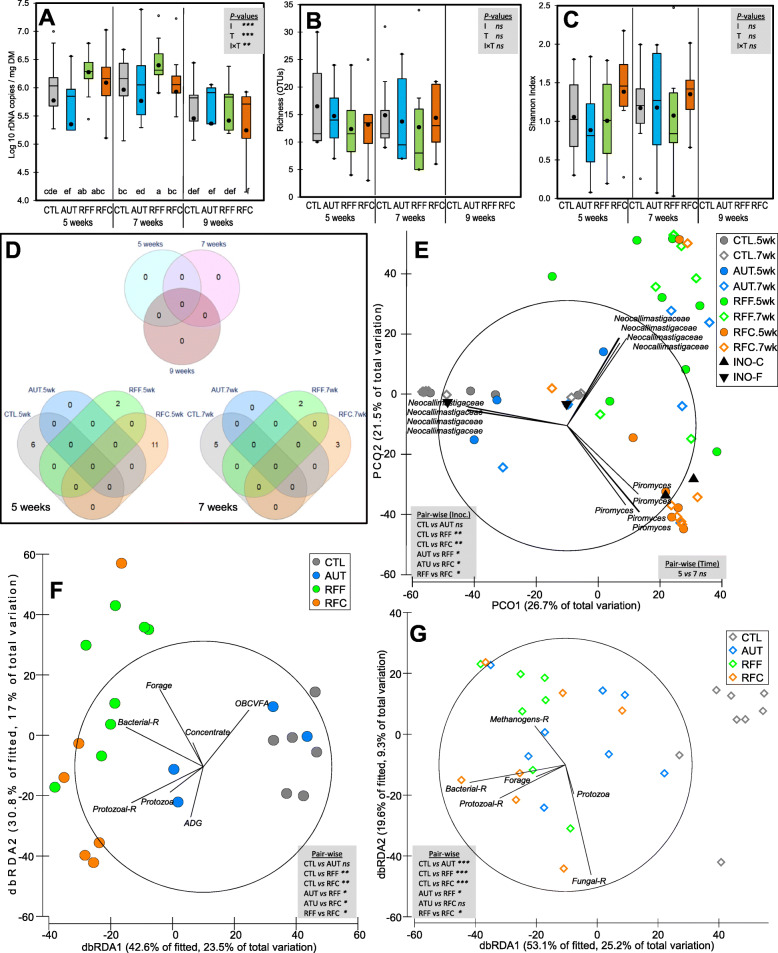
Effects of early-in-life rumen microbial inoculation on the anaerobic fungal community in goats. Boxplots indicating the total anaerobic fungi abundance (**A**) and diversity indexes (**B,C**). Venn diagrams indicating the number of OTUs in the core community (**D**). Principal co-ordinates analysis (**E**) illustrating the treatment effects on the anaerobic fungal community showing the most discriminant OTUs (*ρ* > 0.75). Distance-based redundancy analysis illustrating relationship between the structure of the anaerobic fungal community and rumen function indicators before (**F**), during (**G)** and after (**H**) weaning. Pair-wise PERMANOVA values are provided in grey boxes based on the Bray-Curtis dissimilarity. Goat kids inoculated (I) with fresh rumen fluid from adult goats adapted to forage-rich (RFF) or concentrate-rich diets (RFC), autoclaved rumen fluid (AUT) or absence of inoculation as control (CTL) and sampled at different times (T). In Figs. A, B and C, treatment means with different letters differ

Inoculation with fresh rumen fluid increased the anaerobic fungal concentration at week 5 in comparison to CTL and AUT kids (Fig. 7a), some of these differences persisted at weaning but disappeared after weaning (interaction, *P* < 0.001). Fungal sequencing yielded an average of 6548 ± 618 high quality sequences per sample for those taken at 5 and 7 weeks. However, the number of reads observed at 9 weeks was unexpectedly low and this time point was not further considered. No differences were found in the anaerobic fungal diversity across treatments (Fig. 7b and c).

An absence of a core anaerobic fungal community was observed since no OTUs were shared across treatments and time points (Fig. 7d). Despite that, a treatment-specific core community was observed in CTL (6 and 5 OTUs at week 5 and 7, respectively), RFF (2 and 2 OTUs) and RFC (11 and 3 OTUs) but not in AUT kids. PERMANOVA revealed that the fungal community structure was highly affected by the inoculation (*P* < 0.001) explaining 29.8% of the total variation, whereas no effect was observed for the age of the kids (Table 1). PCoA analysis (Fig. 7e) showed a clear separation between kids inoculated with fresh rumen fluid (right) and those from CTL and AUT (left). A different fungal community was observed between RFF (top) and RFC samples (bottom) being these latter samples correlated with the presence of several *Piromyces* OTUs.

Inoculation with rumen fluid greatly modified the fungal colonization process in terms of taxa abundance (Fig. 6b and Suppl. Table S6). CTL kids had an anaerobic fungal community composed by only 3 taxa: *Neocallimastigaceae* spp. (76%), *Neocallimastix* (10%) and *Caecomyces* (14%). AUT kids showed lower numbers of *Neocallimastigaceae* spp. and *Neocallimastix* than CTL kids, but higher numbers of *Caecomyces*, *Piromyces*, *Orpinomyces* and *Capnodiales*. Moreover, RFF showed the highest abundance of *Orpinomyces*, whereas RFC did so for *Piromyces*. Correlation analysis showed that *Caecomyces* negatively correlated with the presence of protozoa (Table 2), whereas *Orpinomyces* positively correlated with indicators of the rumen physiological (concentrate and DM intakes) and microbiological development (higher bacterial and protozoal richness). *Piromyces* was correlated with even more indicators of the rumen microbiological development (including methanogens and protozoal concentrations and methanogens richness). The higher abundance of *Pyromices* observed in RFC inocula than in RFF inocula was also reflected in RFC kids.

## Discussion

In our study two types of fresh rumen inocula, along with autoclaved inocula, were used to test whether the microbes (or their fermentation products) could affect rumen microbial colonization in early life as previously suggested [20, 21]. Overall, the inoculation with fresh rumen fluid accelerated the rumen microbial development in goat kids resulting in a more complex and diverse microbiota which facilitate the transition from liquid to solid feeding and minimized the weaning stress. However, this intervention had different impact on the main rumen microbial communities as further discussed.

### Rumen bacterial community

The rumen bacterial colonization is a sequential process in which *Proteobacteria* represent the main early colonizers, followed by increasing complexity and abundances of strictly anaerobes such as *Bacteroidetes* and *Firmicutes* [22]. Our study showed that inoculation with fresh rumen fluid accelerated this colonization process during the pre-weaning period leading to a higher diversity (+ 234 OTUs), and a greater core bacterial community shared between RFF and RFC kids (+ 117 OTUs) than reported in CTL kids. Our study showed that CTL kids had higher abundance of several *Proteobacteria* families during the pre-weaning period (e.g. *Comamonadaceae* and *Pasteurellaceae*) indicating delayed microbial colonization. On the contrary, RFF and RFC kids had a higher abundance of members of the *Succinivibrionaceae* family (e.g. *Ruminobacte*r, *Succinomonas* and *Succinivribrio*). This latter family is reported to become more predominant when starter concentrate is consumed [6] due to its ability to degrade starch and simple sugars into propionate [23, 24], as reported here. The positive correlations observed with concentrate intake, propionate, butyrate and ADG confirm the importance of *Succinivibrionaceae* in the transition from milk to solid feed [21] and in the rumen functional development [25].

An increase in the ratio *Firmicutes* to *Bacteroidetes* has been reported when animals consume forage diets [5, 26], and a decrease in the abundance of *Bacteroidetes* has been described when animals start eating solid feed [6], and both aspects are confirmed in our study. In particular, CTL kids, in comparison to those inoculated with fresh rumen fluid, had higher abundance of several *Bacteroidetes* families such as *Bacteroidales* (42% vs 15%) and *Bacteroidaceae* (7.6% vs 0%) during the pre-weaning period. These bacterial taxa were negatively correlated with feed intake, rumen microbial complexity and ADG during the post-weaning period confirming that they are indicators of an underdeveloped rumen bacterial community, as previously reported [16]. On the contrary, RFF and RFC kids had increased abundances of key *Firmicutes* families such as *Veillonellaceae* (+ 2.78 folds) and *Acidaminococcaceae* (+ 2.46 folds), the plant degrader *Rikenellaceae* (+ 7.28 folds) during the pre-weaning period, as well as the presence of *Fibrobacter* which is considered a highly specialized cellulose and hemicellulose degrader [27]. These microbiological changes are signs of a greater maturity of their bacterial community since all these taxa are strict anaerobes capable of degrading fibrous recalcitrant substrates [28, 29] and showed positive correlation with the forage intake and ADG during the post-weaning period. Several other minor phyla such as *Chloroflexi*, *Cyanobacteria*, *Elusimicrobia*, *Spirochaetes* and *Tenericutes* had also higher abundance in kids inoculated with fresh rumen fluid. *Spirochaetes* is considered a xylan degrader, whereas *Cyanobacteria* is most probably *Melainabacteria* which is considered an anaerobic microbe able to act as hydrogen producer [30]. This inoculation with rumen fluid allowed the rumen colonization by *Elusimicrobia, Anaerobiospirillum, Catenisphaera, Clostridiaceae, Marvinbryantia, Saccharofermentans, Quinella, Selenomonas, Rhodocyclaceae, Succinomonas, Synergistes, Olicosphaeraceae, PeH15* and *SP3-e08*, which are mostly strict anaerobes and are considered late rumen colonizers involved in solid feed degradation [16] and were not present in CTL kids during the pre-weaning period. Therefore, their presence in the rumen can explain the greater bacterial diversity, redundancy and adaptability to solid feed observed in RFF and RFC animals [31]. Similar increase in diversity and solid feed intake was reported in artificial-suckling calves with early feeding with solid feed in comparison to those fed milk until weaning [32]. Therefore, our results are in agreement with previous suggestions indicating that rumen bacterial programming through inoculation of rumen fluid is possible [7] with the effects persisting, to some extent, during the post-weaning period since CTL bacterial community remained less developed before and after weaning.

In an earlier in vitro study we evaluated the microbial activity of different types of rumen inocula, sampling time, and preservation methods. It was concluded that fresh rumen fluid sampled at 3 h after feeding provides the most diverse and active inoculum [18]. The present in vivo study builds upon these observations since it has been suggested that the use of microbiologically divergent inocula could modulate the establishment and shape of the rumen bacterial community composition [15, 33]. Our study did not agree with this hypothesis with regards to the bacterial community since most of the differences found between RFF and RFC inocula were not reflected in the inoculated kids. This observation suggests that the bacterial community structure present in the inocula is not that relevant, since only those microbes able to colonize the newborn animal will get established [34]. As a result, as time progressed the bacterial community structure became more similar between all three groups of inoculated kids, probably because of a complete establishment of an adult-like bacterial community after weaning, when animals fed on solid feeds [32] and as a result of the appearance of rumen protozoa in AUT kids as described below. This observation agrees with most studies in which only the bacterial community was studied [15] concluding that inoculation of young ruminants with rumen fluid is more effective when performed before than after weaning [16].

### Rumen methanogens community

Although there are no publications describing the effect of early microbial inoculation on the rumen methanogens and protozoal communities, it has been observed that the natural microbial transfer that occurs by the contact with adult animals promotes substantial changes in the methanogens [1] and protozoal communities [35]. Our study revealed that inoculation with fresh rumen fluid accelerated the rumen colonization by methanogens during the pre-weaning period leading to higher concentrations (+ 2.0 log10 units), richness (+ 5.5 OTUs) and core community, and promoting a more mature methanogens community than observed in CTL kids. This might be caused by the acquisition of protozoa-associated methanogens, which stands for approximately 20% of the methanogens [36], as it has been shown that presence of protozoa increases methanogens diversity [37].

Skillman et al. [2] reported that methanogens colonize the rumen in the first 2 weeks of life. We noted that the most abundant genus across all samples was *Methanobrevibacter* which was identified as a member of the core community as previously described [21, 38]. *M. gottschalkii*, a highly abundant hydrogenotrophic species [39], was predominant in CTL kids before weaning, whereas the inoculation with fresh rumen fluid promoted the proliferation of other methanogens such as *Methanomassiliicocaccaceae* spp., *M. mobile*, *M. ruminantium* and Group9_sp, promoting a more diverse community. These latter taxa showed positive correlations with indicators of the rumen microbiological development (protozoal concentration and richness) and physiological development (higher forage intake and ADG during the post-weaning period) possibly because some of them (e.g. *Methanobrevibacter* and *Methanomicrobium*) have been found inside of rumen protozoa (endosymbionts) and can favour the inter-species H_2_ transport and ultimately feed utilization [40]. Similarly, *M. ruminantium* requires the presence of other methanogen species to thrive; therefore its presence can be considered an indicator of rumen microbial maturity [2, 5]. Most of the differences in the methanogens community observed during the pre-weaning period (e.g. higher diversity and abundance of *M. ruminantium*) were maintained during the weaning and the post-weaning stages. As a result, CTL kids still lacked relevant taxa such as *Methanosphaera, M. blatticola* and some *Methanomassilicoccaceae* members, indicating an underdeveloped methanogens community in comparison to RFF and RFC kids. A greater methanogens core community and diversity was also observed at weaning in lambs under natural vs artificial milk feeding [41] indicating that inoculation with fresh rumen fluid can partially mimic the microbial transfer from the dam to the offspring.

The positive effect of the inoculation with fresh rumen fluid was more evident for RFF than for RFC, possibly as a result of the higher methanogens OTU richness in the RFF inoculum. This observation suggests that the concept of “rumen microbial programming” of the methanogens community based on the modification of the colonization process should not be ruled out [7]. The higher methanogens concentration and diversity observed in AUT than CTL kids could be an indirect effect mediated by the presence of protozoa as described below [37].

### Rumen protozoal community

Anaerobic protozoa are late rumen colonizers because they are highly sensitive to oxygen and require direct contact between young and adult ruminants for an effective transmission, drinking water being the most likely mode of transfer [42]. This lack of contact with adult ruminants justifies the absence of protozoa in CTL kids during the entire duration of the study, an aspect that we have also described previously in artificially reared lambs [41]. A natural sequence of rumen colonization has been described for the different protozoal families starting by *Entodiniinae,* followed by *Diplodiniinae* and *Ophyoxcolecinae* and finishing with holotrichs [42]. The visual microscopy examination of the protozoal community [19] and sequencing data confirmed this sequence. The presence of a small concentration of protozoa, mostly composed of *Entodinium,* in AUT kids located in three contiguous pens suggested that they may have accidentally been cross-faunated before week 5. Moreover, the lack of holotrichs and *Diplodinium* at week 5 indicated an incomplete and delayed rumen protozoal colonization in AUT kids given the inherent difficulty of holotrichs to become stabilized in the rumen of young ruminants [43, 44]. This partial faunation of the AUT kids, along with the potential positive effects of some metabolites present in the autoclaved rumen fluid (e.g. VFA, microbial extracts, micro-nutrients) in young calves [20, 21], could explain the moderate but positive impact of this treatment on the rumen microbial and physiological development noted in our study.

Our findings clearly indicated that inoculation with fresh rumen fluid accelerated the rumen protozoal colonization during the pre-weaning period both in concentration and diversity (+ 27 OTUs). As a result, RFF and RFC kids had increased number of fibrolytic protozoa (*Diplodinium, Ophryoscolex* and *Enoploplastron*) and holotrichs (*Isotricha* and *Dasytricha*) which are considered late rumen colonizers involved in the H_2_ production [45]. The results suggested that these protozoal taxa, along with the protozoal richness, can be considered indicators of the rumen microbial and functional development. The symbiotic relations between rumen protozoa and methanogens in relation to the inter species H_2_ transfer [46], and between protozoa and bacteria in relation to cross-feeding processes [45] could partially explain the positive correlation of these protozoal taxa with methanogens and bacterial diversity, as well as with solid feed intake. Moreover, it has been suggested that bacterial predation by protozoa might stimulate the proliferation of different bacterial species occupying similar metabolic niches [47]. After conducting a meta-analysis, Newbold et al. [35] concluded that presence of rumen protozoa (in comparison to defaunated animals) have a positive effect on feed intake (+ 2%), VFA production (+ 5%) and NDF digestibility (+ 11%) but negative on the microbial protein synthesis (− 30%) and ADG (− 9%) being these differences diet-dependent. Our findings suggested that the early colonization of the rumen by a mature protozoal community had positive effects under artificial-milk feeding conditions facilitating the transition from liquid to solid feed.

As it was expected, RFF and RFC kids had a similar protozoal community structure and shared a large core community (up to 23 OTUs), due to the lack of substantial differences in the protozoal community between both types of fresh inocula. However, the protozoal community structure in AUT kids also converged with that observed in RFF and RFC kids during the post-weaning period. These findings suggest that the rumen protozoal colonization process can be modulated by the type of microbial inocula, but the persistency of the those differences is weak and kids tend to converge into a similar protozoal community, possibly as a result of a cross-faunation between animals [48].

### Rumen fungal community

Rumen fungi are considered late rumen colonizers [49]. Like rumen protozoa, anaerobic fungi are high sensitive to oxygen, but their ability to form resistant spores allows them to retain viability in dung, soil and feed, making their transmission easier [50]. Fonty et al. [51] found anaerobic fungi (mostly *Neocallimastix*) in the rumen of flock-reared lambs by 8–10 days after birth, although their presence was intermittent and highly variable until weaning. Orpin [52] also reported that fungi are apparently able to colonize the rumen before the ingestion of large amounts of solid feed. Our findings support this hypothesis since an abundant and diverse anaerobic fungal community was observed before weaning across all treatments. CTL kids were particularly abundant on uncultured *Neocallimastigaceae* genera but lacked some of the most relevant genera such as *Piromyces, Orpinomyces* and *Capnodiales*, indicating the presence of an underdeveloped and treatment-specific fungal core community. On the contrary, inoculation with fresh rumen fluid promoted a more abundant and different fungal community than observed in CTL and AUT kids before weaning. In a previous study we showed higher fungal diversity in naturally reared lambs (with their dams) than in their artificially reared counterparts at weaning [41], differences which were not noted in the present study. Instead, inoculation with fresh rumen fluid modulated the fungal community structure during the pre-weaning period leading to increased numbers of *Piromyces* and *Orpinomyces*. *Piromyces* has been described to degrade a wide range of plant structural materials as well as glucose [53, 54] and it has been correlated with ADG during the post-weaning period [41]. *Orpinomyces* is considered a relevant cellulose and xylanase degrader [55] particularly abundant in grazing lambs [5]. Our study showed that *Piromyces* was positively correlated with the bacterial, methanogens and fungal diversities and was the main signature associated to RFC inocula and RFC kids. On the other hand, whereas *Orpinomyces* correlated with solid feed intake indicating that both are indicators of the rumen development.

It has been suggested that once animals start eating solid feed, the feed composition is the main determining factor of the rumen fungal community [52]. Our study did not allow discerning whether the observed changes in the fungal community were directly determined by the inoculating process or indirectly by the increase in the solid feed intake. Similar co-occurring effects (higher solid feed intake and higher fungal development) were reported in young lambs fed natural milk feeding, in comparison to natural reared, with the peculiarity that these effects persisted during the grazing period leading to higher forage digestibility and ADG during later in life [41]. Our study seems to agree with these findings because RFF and RFC showed higher forage intake than CTL kids up to week 13, but the persistency of the effects on the fungal community needs further research.

### Rumen microbiota and animal performance

In terms of productive outcomes, the overall acceleration of the rumen microbial colonization induced by the inoculation with fresh rumen fluid positively correlated with a concomitant acceleration in the rumen functional development during the pre-weaning period. This development implied higher solid feed intake, rumen VFA and blood β-hydroxybutyrate concentrations than in CTL kids [19]. Despite the aforementioned positive indicators, inoculation with fresh rumen fluid did not improve ADG as reported in previous studies [15, 16, 56], possibly because the more complex microbiota is associated with lower feed efficiency when ruminants are fed concentrated diets [31]. However, in our study, this microbial complexity and redundancy provided higher adaptability. As a result, kids inoculated with fresh rumen fluid experienced a higher ADG at week 8 (immediately after weaning), indicating that these kids experienced less growth retardation and weaning shock resulting on positive health and welfare outcomes [19]. Our meta-taxonomic study did not allow assessing causality between this acceleration in the microbial development and changes and its function. The use of metagenomics shotgun sequencing could help to bridge this gap by facilitating a direct inference of the microbial functionality potential.

## Conclusions

This experiment based on a multi-kingdom analysis of the rumen microbiome revealed that an early-in-life repetitive inoculation of young ruminants with fresh rumen fluid from adult animals accelerated the establishment of a more complex and diverse bacterial, methanogenic, protozoal and fungal communities during the pre-weaning period. This microbial complexity facilitated the adaptability of the host ruminant to nutritional challenges favoring the transition from milk to solid feeding during the weaning process. The intensity and persistency of the microbiological effects varied depending on the rumen microbial community considered. The type of diet consumed by the donor animal promoted substantial differences in the inocula, however those microbiological differences were mostly not reflected in the inoculated kids resulting on similar productive outcomes and suggesting that alternative factors such as the availability of nutrients for the rumen microbes or the host-immune system may play a relevant role during the rumen microbial colonization. The inoculation of autoclaved rumen fluid also promoted positive effects on the rumen function but much less evident than using fresh inocula. Further research is needed to evaluate the persistency of these effects in adult animals and their impact on animal productivity.

## Methods

### Description of the inocula

Animal procedures were conducted by trained personnel according to the Spanish guidelines (RD 53/2013), and protocols were approved by the Ethical Committee for Animal Research (EEZ-CSIC) regional government (09/03/2017). As described in [19], eight adult Murciano-Granadina goats with permanent rumen fistula were distributed into two groups and used as rumen fluid donors. Four received a 100% forage-based diet consisting in 750 g alfalfa hay and 750 g oat hay daily, whereas the other four were fed a concentrate-based diet consisting in 800 g concentrate feed, 125 g alfalfa hay and 125 g oat hay. The forage chemical composition (in g/kg DM) was organic matter 906, nitrogen 25, neutral detergent fiber 594, acid detergent fire 366, acid detergent lignin 95 and ether extract 16, while the pelleted concentrate (Lactación Rumiantes, Macob, Granada, Spain) was 951, 33, 254, 73, 21 and 45, respectively. Diet was offered at 1.2 times maintenance level and divided into two equal meals (8:00, 16:00 h).

After two weeks of adaptation to the diet, rumen fluid from donor goats fed forage (RFF) or concentrate diets (RFC) were collected daily 3 h after the morning feeding (100 ml/donor), pooled by diet, strained through a cheesecloth (approx. 1 mm pore size), bubbled with CO_2_, maintained at 37 °C in a pre-warmed thermal flask and immediately administered as fresh inoculum to young kids. Autoclaved inoculum (AUT) was prepared weekly by mixing equal volumes of RFF and RFC inocula from all donors and autoclaved at 115 °C for 30 min to destroy all microbes but maintaining the rumen fermentation products. Four subsamples from each type of inocula were taken at regular intervals for inocula characterization (Suppl. Table S1).

### Inoculation experiment

A total of 80 newborn Murciano-Granadina goat kids were randomly distributed in 4 experimental groups: RFF and RFC kids were inoculated with rumen fluid from adult goats fed forage-rich or concentrate-rich diets, respectively. AUT kids were inoculated with the autoclaved rumen fluid whereas CTL kids received no inoculation. Inoculation consisted of an oral and daily drench of rumen fluid (2.5 ml/animal during week 1 and 5 ml/animal thereafter) from day 1 until 11 weeks of age. Kids from different treatments were separated by a 2-m-wide corridor to prevent physical contact. Inoculation was performed by trained personnel following always the same sequence (AUT followed by RFF and RFC kids) and changing all inoculation material (e.g. drench and gloves) to prevent cross-contamination between experimental groups.

After parturition, all kids were separated from their mother and received approximately 200 ml of pooled natural colostrum divided in two doses. To avoid any initial bias, average body weight (BW) and males/females ratio was kept similar in all treatments and siblings were always allocated into different treatments. Kids within each treatment were distributed in 5 contiguous pens with similar age (maximum 2 days difference) and were handled and sampled on the same day across treatments.

All kids were raised on commercial milk replacer (Univet Spray, Cargill, Barcelona, Spain) offered ad libitum. From week 2, kids had free access to the same forage mixture that has been described for the donor goats and to pelleted starter concentrate (0–14 Rumiantes Transición, Macob, Granada, Spain) with the following chemical composition (in g/kg DM): 949 OM, 226 CP, 319 NDF, 87 ADF, 34 ADL, 48 EE. Kids were weaned at 7 weeks of age by progressively decreasing milk powder concentration during 4 days. Forage and concentrate intakes were daily recorded, BW, blood glucose and β-hydroxybutyrate concentrations were weekly monitored.

### Rumen microbial sampling and analyses

Rumen microbiota was studied at weeks 5 (pre-weaning), 7 (weaning) and 9 (post-weaning). Rumen content was withdrawn by orogastric intubation at 09:00 h as previously described [19]. Rumen samples (ca. 50 ml) were filtrated through sterile cheesecloth (approx. 1 mm pore size) and all sampling instruments were changed between animals to prevent cross contamination. A subsample of rumen fluid was snap-frozen in liquid N whereas solids were discarded given the small and variable proportion of solids in the samples. Rumen samples were freeze-dried, bead-beated for 1 min (MiniBeadBeater, Biospect Products, Bartlesville, OK, USA) and DNA was extracted using a commercial kit (QIAamp DNA Stool Mini Kit, Qiagen Ltd., Barcelona, Spain). DNA was also extracted from the rumen fluids used as inocula (positive controls) and from negative controls (DNA extraction without rumen fluid) and further analyzed. Eluted DNA (2 μl) was used to assess the abundance of the main microbial groups by quantitative PCR (qPCR) an iQ5 multicolor Real-Time PCR Detection System (BioRad Laboratories Inc., Hercules, CA, USA). Specific primers for the 16S bacterial rRNA gene, mcrA gene for archaea and 18S rRNA genes for protozoa and anaerobic fungi were used as reported in Supplementary Table S8. Rumen fermentation characteristics in terms of pH, ammonia and volatile fatty acids (VFA) was also described at the same ages [19]. This metadata (Suppl. Table S7) was used to relate microbial changes in the rumen with animal physiology.

### Next generation sequencing

For meta-taxonomic analyses 8 kids from each treatment were selected (all males from pens 1, 2, 3 and 4) and a template of rumen DNA was sent to University of Illinois Biotechnology Center (Urbana, IL, USA) for Fluidgim amplicon sequencing using Miseq V3 (Illumina Inc., San Diego, CA, USA). Primers used for the amplification of the bacterial 16S (V3-V5 region), methanogens 16S, protozoal 18S and anaerobic fungal ITS3-ITS4 regions were used as described in Supplementary Table S8.

For each of the 4 major microbial groups, primer sorted and demultiplexed paired-end reads were merged and then combined into one file. Downstream processing was performed using QIIME [57] and Mothur [58] for archaea, PIPITS [59] for fungi and IM-Tornado [60] for bacteria and protozoa, where non-overlapping reads are processed while retaining maximal information content. Low-quality reads and bases (PHRED quality score below 25) were trimmed. Minimum length of reads after quality filtering was 350 for bacteria and archaea, 187 for protozoa and 300 for fungi. Chimeras were identified and removed using chimera.vsearch [61]. Operational taxonomic units (OTU) were identified at 97% similarity level and then representative sequences from all OTUs were aligned against Greengenes 13_8 97% [62] for bacteria, RIM-DB [63] for archaea, Silva v. 132 [64] for protozoa and UNITE [65] for fungi. Once alignment was performed, data from each of the 4 major microbial groups were processed separately. The number of sequences per sample for each microbial group was normalized across all the samples and singletons were removed. Only sequences from rumen protozoa *(subclass Trichostomatia*) and anaerobic fungi (class *Neocallimastigomycota*) were further considered to prevent potential bias derived from transient or non-rumen eukaryotes [66]. Raw sequences reads were deposited at European Nucleotide Archive repository (accession: ERP122902).

### Calculations and statistical analyses

Statistical analyses were conducted using SPSS software (IBM Corp., Version 21.0, New York, USA). Quantitative PCR data (rDNA copies/mg DM) and taxa abundances (sequences) were tested for normality using the Shapiro–Wilk test and data were log10 transformed to achieve a normal distribution. Data were analysed based on a repeated measures mixed –effects (residual maximum likelihood) as follows:

$$ {Y}_{ij kl}\kern1.25em =\mu +{I}_i+{T}_j+{\left(I\times T\right)}_{ij}+{G}_k+A{(G)}_l+{e}_{ij kl} $$

where *Y*_*ijkl*_ is the dependent, continuous variable, *μ* is the overall population of the mean, *I*_*i*_ is the fixed effect of the inoculation (*i* = CTL vs AUT vs RFF vs RFC), *T*_*j*_ is the fixed effect of the sampling time or age (*j* = 5 vs 7 vs 9 weeks), *(I × A)*_*ij*_ is the interaction term, *G*_*k*_ is the random effect of the pen considered as a block (*j* = 1 to 5), *A(G)*_*l*_ is the random effect of the animal nested to the pen (*l* = 1 to 80 for qPCR and 1 to 32 for sequencing data) and *e*_*ijkl*_ is the residual error. For taxonomic data, False Discovery Rate was minimized using the Bonferroni statistical. Significant effects were declared at *P* < 0.05, tendency to difference at *P* < 0.1 and abbreviated as follows: *** *P* < 0.001; ** *P* < 0.01; * *P* < 0.05; † *P* < 0.1; ns, not significant.

Venn diagrams were performed to illustrate the treatment effects on the core microbial community which was defined as the number of OTUs shared across the majority (> 75%) of the individuals in each treatment/time [8] using a multiple list comparator (www.molbiotools.com). Treatment effects on the rumen multi-kingdom (including all microbial groups) and on the bacterial, methanogens, protozoal and anaerobic fungal communities were assessed based on the Bray-Curtis distance metrics using the UPGMA function of PRIMER-6 software (PRIMER-E Ltd., Plymouth, UK). Log10-transformed data were analyzed by non-parametric PERMANOVA after 999 random permutations of residuals under the reduced model using the Monte Carlo test [67]. When a significant factor was found in the PERMANOVA, pair-wise comparisons were performed using the same software and settings (999 permutations) to elucidate differences between treatments. Principal Coordinate analysis (PCoA) were performed to illustrate the impact of the treatments on the overall microbial community structure and tripod vectors were included to describe the direction and intensity of the most discriminant OTUs (Spearman’s correlation > 0.55). Given that three different nutritional situations were studied, Distance-Based Redundancy Analyses were performed to illustrate the relationship between the community structure of the rumen microbiota and metadata (32 variables reported in Suppl. Table S2) describing the rumen function and animal performance at 5, 7 and 9 weeks of age. Distance based linear models (DistLM) were developed based on the Bray-Curtis similarity and the predictor variables were selected based on a step-wise procedure with 999 random permutations following the Akaike information criterion with corrections for small sample size (AICc) to avoid model overfitting. Only predictor variables which resulted significant (*P* < 0.05) were included in the final model. Spearman correlations (*ρ)* were calculated to assess the relationships between the microbial taxa abundance (log10 number of sequences) and the metadata. Strong correlations were defined as those with *ρ* ≥ 0.3 or ≤ − 0.3 and *P* < 0.01.

## Supplementary Information

**Additional file 1 Table S1.** Description of the inocula in terms of rumen fermentation and microbial composition. **Table S2.** Effect of the early-in-life rumen microbial inoculation and age on the rumen bacteria concentration, diversity and taxonomy. **Table S3.** Spearman’s correlations between the bacterial taxa and the rumen function and animal performance. **Table S4.** Effect of early-in-life rumen microbial inoculation and age on the rumen methanogens concentration, diversity and taxonomy. **Table S5.** Effect of early-in-life rumen microbial inoculation and age on the rumen protozoal concentration, diversity and taxonomy. **Table S6.** Effect of early-in-life rumen microbial inoculation and age on the rumen anaerobic fungal concentration, diversity and taxonomy. **Table S7.** Descriptive statistics of the metadata used in the distance-based redundancy analyses and spearman correlations with microbial taxa abundance. **Table S8.** Primers used for quantitative PCR and Next Generation Sequencing. **Fig. S1**. Principal co-ordinates analysis illustrating the inoculation effects on the multi-kingdom rumen microbioma.

## Data Availability

All sequencing data generated in this study are publicly available in the European Nucleotide Archive repository (accession: ERP122902).

## Abbreviations

ADG: Average daily gain
AUT: Goat kids inoculated with autoclaved rumen fluid
BHB: Beta-hydroxybutyrate
BW: Body weight
CTL: Control goat kids without inoculation
dbRDA: Distance-based redundancy analysis
DCI: Digestible cellulose intake
DHCI: Digestible hemicellulose intake
PCoA: Principal Coordinate Analysis
PERMANOVA: Permuntational analysis of variance
RFC: Goat kids inoculated with rumen fluid adapted to concentrate diet
RFF: Goat kids inoculated with rumen fluid adapted to forage diet
VFA: Volatile fatty acids

## References

[1] Abecia L, Ramos-Morales E, Martínez-Fernandez G (2014). Feeding management in early life influences microbial colonisation and fermentation in the rumen of newborn goat kids. Anim Prod Sci.

[2] Skillman LC, Evans PN, Naylor GE, Morvan B, Jarvis GN, Joblin KN (2004). 16S ribosomal DNA-directed PCR primers for ruminal methanogens and identification of methanogens colonising young lambs. Anaerobe..

[3] Davis CL, Drackley JK. The Development, Nutrition, and Management of the Young Calf ,. Iowa State.; 1998.

[4] Baldwin VIRL, McLeod KR, Klotz JL, Heitmann RN (2004). Rumen development, intestinal growth and hepatic metabolism in the pre- and Postweaning ruminant. J Dairy Sci.

[5] Belanche A, Kingston-Smith AH, Griffith GW, Newbold CJ (2019). A multi-kingdom study reveals the plasticity of the rumen microbiota in response to a shift from non-grazing to grazing diets in sheep. Front Microbiol.

[6] Rey M, Enjalbert F, Combes S, Cauquil L, Bouchez O, Monteils V (2014). Establishment of ruminal bacterial community in dairy calves from birth to weaning is sequential. J Appl Microbiol.

[7] Yáñez-Ruiz DR, Abecia L, Newbold CJ (2015). Manipulating rumen microbiome and fermentation through interventions during early life: a review. Front Microbiol.

[8] Furman O, Shenhav L, Sasson G (2020). Stochasticity constrained by deterministic effects of diet and age drive rumen microbiome assembly dynamics. Nat Commun.

[9] Nylund L, Satokari R, Salminen S, De Vos WM (2014). Intestinal microbiota during early life - impact on health and disease. Proc Nutr Soc.

[10] Yeoman CJ, Ishaq SL, Bichi E, Olivo SK, Lowe J, Aldridge BM (2018). Biogeographical differences in the influence of maternal microbial sources on the early successional development of the bovine neonatal gastrointestinal tract. Sci Rep.

[11] Alipour MJ, Jalanka J, Pessa-Morikawa T (2018). The composition of the perinatal intestinal microbiota in cattle. Sci Rep.

[12] Lu CD, Potchoiba MJ (1988). Milk feeding and weaning of goat kids - a review. Small Rumin Res.

[13] Abecia L, Jiménez E, Martínez-Fernandez G (2017). Natural and artificial feeding management before weaning promote different rumen microbial colonization but not differences in gene expression levels at the rumen epithelium of newborn goats. PLoS One.

[14] De Barbieri I, Hegarty RS, Silveira C, Oddy VH (2015). Positive consequences of maternal diet and post-natal rumen inoculation on rumen function and animal performance of merino lambs. Small Rumin Res.

[15] De Barbieri I, Hegarty RS, Silveira C (2015). Programming rumen bacterial communities in newborn merino lambs. Small Rumin Res.

[16] Yu S, Zhang G, Liu Z, Wu P, Yu Z, Wang J (2020). Repeated inoculation with fresh rumen fluid before or during weaning modulates the microbiota composition and co-occurrence of the rumen and colon of lambs. BMC Microbiol.

[17] Huws SA, Creevey CJ, Oyama LB (2018). Addressing global ruminant agricultural challenges through understanding the rumen microbiome: past, present, and future. Front Microbiol.

[18] Belanche A, Palma-Hidalgo JM, Nejjam I (2019). In vitro assessment of the factors that determine the activity of the rumen microbiota for further applications as inoculum. J Sci Food Agric.

[19] Belanche A, Palma-Hidalgo JM, Nejjam I, Jiménez E, Martín-García AI, Yáñez-Ruiz DR (2020). Inoculation with rumen fluid in early life as a strategy to optimize the weaning process in intensive dairy goat systems. J Dairy Sci.

[20] Muscato TV, Tedeschi LO, Russell JB (2002). The effect of ruminal fluid preparations on the growth and health of newborn, milk-fed dairy calves. J Dairy Sci.

[21] O’Hara E, Kelly A, McCabe MS, Kenny DA, Guan LL, Waters SM (2018). Effect of a butyrate-fortified milk replacer on gastrointestinal microbiota and products of fermentation in artificially reared dairy calves at weaning. Sci Rep.

[22] Jami E, Israel A, Kotser A, Mizrahi I (2013). Exploring the bovine rumen bacterial community from birth to adulthood. ISME J.

[23] O’Herrin SM, Kenealy WR (1993). Glucose and carbon dioxide metabolism by Succinivibrio dextrinosolvens. Appl Environ Microbiol.

[24] Danielsson R, Dicksved J, Sun L (2017). Methane production in dairy cows correlates with rumen methanogenic and bacterial community structure. Front Microbiol.

[25] Li F, Guan LL (2017). Metatranscriptomic profiling reveals linkages between the active rumen microbiome and feed efficiency in beef cattle. Appl Environ Microbiol.

[26] Fernando SC, Purvis HT, Najar FZ (2010). Rumen microbial population dynamics during adaptation to a high-grain diet. Appl Environ Microbiol.

[27] Ransom-Jones E, Jones DL, McCarthy AJ, McDonald JE (2012). The Fibrobacteres: an important phylum of cellulose-degrading Bacteria. Microb Ecol.

[28] Biddle A, Stewart L, Blanchard J, Leschine S (2013). Untangling the genetic basis of fibrolytic specialization by lachnospiraceae and ruminococcaceae in diverse gut communities. Diversity..

[29] Brulc JM, Antonopoulos DA, Berg Miller ME (2009). Gene-centric metagenomics of the fiber-adherent bovine rumen microbiome reveals forage specific glycoside hydrolases. Proc Natl Acad Sci U S A.

[30] Di Rienzi SC, Sharon I, Wrighton KC (2013). The human gut and groundwater harbor non-photosynthetic bacteria belonging to a new candidate phylum sibling to cyanobacteria. Elife..

[31] Kruger Ben Shabat S, Sasson G, Doron-Faigenboim A, et al. Specific microbiome-dependent mechanisms underlie the energy harvest efficiency of ruminants. ISME J. 2016;10:2958–72. 10.1038/ismej.2016.62 PMC5148187 27152936

[32] Dias J, Marcondes MI, Noronha MF (2017). Effect of pre-weaning diet on the ruminal archaeal, bacterial, and fungal communities of dairy calves. Front Microbiol.

[33] Dehority B. Rumen Microbiology: Nottingham University Press; 2003.

[34] Friedman N, Jami E, Mizrahi I (2017). Compositional and functional dynamics of the bovine rumen methanogenic community across different developmental stages. Environ Microbiol.

[35] Newbold CJ, De la Fuente G, Belanche A, Ramos-Morales E, McEwan NR (2015). The role of ciliate protozoa in the rumen. Front Microbiol.

[36] Mackie R, McSweeney C, Klieve A. 4 Microbial Ecology of the Ovine Rumen. Sheep Nutr. Published online 2002:71.

[37] Belanche A, De La Fuente G, Newbold CJ (2015). Effect of progressive inoculation of fauna-free sheep with holotrich protozoa and total-fauna on rumen fermentation, microbial diversity and methane emissions. FEMS Microbiol Ecol.

[38] Zhou M, Chen Y, Griebel PJ, Guan LL (2015). Methanogen prevalence throughout the gastrointestinal tract of pre-weaned dairy calves. Gut Microbes.

[39] Tapio I, Snelling TJ, Strozzi F, Wallace RJ (2017). The ruminal microbiome associated with methane emissions from ruminant livestock. J Anim Sci Biotechnol.

[40] Sharp R, Ziemer CJ, Stern MD, Stahl DA (1998). Taxon-specific associations between protozoal and methanogen populations in the rumen and a model rumen system. FEMS Microbiol Ecol.

[41] Belanche A, Yáñez-Ruiz DR, Detheridge AP, Griffith GW, Kingston-Smith AH, Newbold CJ (2019). Maternal versus artificial rearing shapes the rumen microbiome having minor long-term physiological implications. Environ Microbiol.

[42] Fonty G, Senaud J, Jouany JP, Gouet P (1988). Establishment of ciliate protozoa in the rumen of conventional and conventionalized lambs: influence of diet and management conditions. Can J Microbiol.

[43] Belanche A, Balcells J, de la Fuente G, Yañez-Ruíz DR, Fondevila M, Calleja L (2010). Description of development of rumen ecosystem by PCR assay in milk-fed, weaned and finished lambs in an intensive fattening system. J Anim Physiol Anim Nutr.

[44] Belanche A, Abecia L, Holtrop G (2011). Study of the effect of presence or absence of protozoa on rumen fermentation and microbial protein contribution to the chyme. J Anim Sci.

[45] Williams, A.G., and Coleman GS. The Rumen Protozoa. (Thomas DB, ed.). Springer-Verlag New York Inc.; 1992.

[46] Newbold CJ, Lassalas B, Jouany JP (1995). The importance of methanogens associated with ciliate protozoa in ruminal methane production in vitro. Lett Appl Microbiol.

[47] Belanche A, De la Fuente G, Moorby JM, Newbold CJ (2012). Bacterial protein degradation by different rumen protozoal groups. J Anim Sci.

[48] Bird SH, Hegarty RS, Woodgate R (2010). Modes of transmission of rumen protozoa between mature sheep. Anim Prod Sci.

[49] Orpin CK, Joblin KN. The rumen anaerobic fungi. In: Hobson PN, Stewart C, eds. The Rumen Microbial Ecosystem. Springer. 1997:140–95.

[50] McGranaghan P, Davies JC, Griffith GW, Davies DR, Theodorou MK (1999). The survival of anaerobic fungi in cattle faeces. FEMS Microbiol Ecol.

[51] Fonty G, Gouet P, Jouany J-P, Senaud J (1987). Establishment of the microflora and anaerobic Fungi in the rumen of lambs. Microbiology..

[52] Orpin CG (1984). The role of ciliate protozoa and fungi in the rumen digestion of plant cell walls. Anim Feed Sci Technol.

[53] de Souza WR. Microbial Degradation of Lignocellulosic Biomass. Sustain Degrad Lignocellul Biomass. Tech Appl Commer. 2013;207–247.

[54] Solomon KV, Haitjema CH, Henske JK (2016). Early-branching gut fungi possess large, comprehensive array of biomass-degrading enzymes. Science..

[55] Li XL, Chen H, Ljungdahl LG (1997). Monocentric and polycentric anaerobic fungi produce structurally related cellulases and xylanases. Appl Environ Microbiol.

[56] Zhong RZ, Sun HX, Li GD, Liu HW, Zhou DW (2014). Effects of inoculation with rumen fluid on nutrient digestibility, growth performance and rumen fermentation of early weaned lambs. Livest Sci.

[57] Caporaso JG, Kuczynski J, Stombaugh J (2010). Correspondence QIIME allows analysis of high- throughput community sequencing data intensity normalization improves color calling in SOLiD sequencing. Nat Publ Gr.

[58] Schloss PD, Westcott SL, Ryabin T (2009). Introducing mothur: open-source, platform-independent, community-supported software for describing and comparing microbial communities. Appl Environ Microbiol.

[59] Gweon HS, Oliver A, Taylor J (2015). PIPITS: an automated pipeline for analyses of fungal internal transcribed spacer sequences from the Illumina sequencing platform. Methods Ecol Evol.

[60] Jeraldo P, Kalari K, Chen X (2014). IM-TORNADO: a tool for comparison of 16S reads from paired-end libraries. PLoS One.

[61] Rognes T, Flouri T, Nichols B, Quince C, Mahé F (2016). VSEARCH: a versatile open source tool for metagenomics. PeerJ..

[62] DeSantis TZ, Hugenholtz P, Larsen N (2006). Greengenes, a chimera-checked 16S rRNA gene database and workbench compatible with ARB. Appl Environ Microbiol.

[63] Seedorf H, Kittelmann S, Henderson G, Janssen PH (2014). RIM-DB: a taxonomic framework for community structure analysis of methanogenic archaea fromthe rumen and other intestinal environments. PeerJ..

[64] Quast C, Pruesse E, Yilmaz P (2013). The SILVA ribosomal RNA gene database project: improved data processing and web-based tools. Nucleic Acids Res.

[65] Nilsson RH, Larsson KH, Taylor AFS (2019). The UNITE database for molecular identification of fungi: handling dark taxa and parallel taxonomic classifications. Nucleic Acids Res.

[66] Hobson PN, Stewart CS. The RUmen Microbla Ecosystem. 2nd ed. Springer; 1997.

[67] Belanche A, Newbold CJ, Lin W, Stevens PR, Kingston-Smith AH (2017). A systems biology approach reveals differences in the dynamics of colonization and degradation of grass vs. Hay by rumen microbes with minor effects of vitamin E supplementation. Front Microbiol.

