# Supplementary material for: Inoculation with rumen fluid in early life accelerates the rumen microbial development and favours the weaning process in goats
Source: Anim Microbiome. 2021 Jan 19;3:11. doi: 10.1186/s42523-021-00073-9 (PMC7814744; doi:10.1186/s42523-021-00073-9)
Supplement: Supplementary file 1 — Additional file 1 Table S1. Description of the inocula in terms of rumen fermentation and microbial composition. Table S2. Effect of the early-in-life rumen microbial inoculation and age on the rumen bacteria concentration, diversity and taxonomy. Table S3. Spearman’s correlations between the bacterial taxa and the rumen function and animal performance. Table S4. Effect of early-in-life rumen microbial inoculation and age on the rumen methanogens concentration, diversity and taxonomy. Table S5. Effect of early-in-life rumen microbial inoculation and age on the rumen protozoal concentration, diversity and taxonomy. Table S6. Effect of early-in-life rumen microbial inoculation and age on the rumen anaerobic fungal concentration, diversity and taxonomy. Table S7. Descriptive statistics of the metadata used in the distance-based redundancy analyses and spearman correlations with microbial taxa abundance. Table S8. Primers used for quantitative PCR and Next Generation Sequencing. Fig. S1. Principal co-ordinates analysis illustrating the inoculation effects on the multi-kingdom rumen microbioma. [file 42523_2021_73_MOESM1_ESM.docx]

Animal microbiome, Supplementary material

**Inoculation with rumen fluid in early life accelerates the rumen microbial development and favours the weaning process**

Palma-Hidalgo J.M., Jiménez E., Popova M., Morgavi D.P., Martín-García A.I., Yáñez-Ruiz D.R., Belanche A.

*Corresponding author: Alejandro Belanche E-mail: [a.belanche@csic.es](mailto:a.belanche@csic.es)

**Running title:** Rumen fluid inoculation accelerates microbial development

**Supplementary Table S1.** Description of the inocula in terms of rumen fermentation and microbial composition.

| **Inoculum^1^** | **AUT** | **RFF** | **RFC** | **s.e.d.** | **P-value** |
| --- | --- | --- | --- | --- | --- |
| Fermentation products | | |  |  |  |
| DM (%) | 4.28^ab^ | 2.35^b^ | 5.22^a^ | 0.877 | 0.043 |
| pH | 6.11^b^ | 6.38^a^ | 5.79^c^ | 0.095 | 0.002 |
| Lactate (mM) | 1.86^a^ | 0.55^b^ | 0.85^b^ | 0.328 | 0.017 |
| Ammonia-N (mg/dL) | 8.64 | 7.27 | 10.0 | 1.095 | 0.117 |
| Total VFA (mM) | 120^ab^ | 103^b^ | 134^a^ | 9.990 | 0.055 |
| Acetate (%) | 63.0^b^ | 70.1^a^ | 55.5^c^ | 1.339 | <0.001 |
| Propionate (%) | 23.6^b^ | 18.2^c^ | 29.0^a^ | 1.711 | <0.001 |
| Butyrate (%) | 10.3^b^ | 9.49^c^ | 11.8^a^ | 0.746 | 0.004 |
| Bacterial community | |  |  |  |  |
| Concentration (log10 copies/l) | | 9.96 | 11.8 | 0.884 | 0.081 |
| Richness |  | 502 | 396 | 30.30 | 0.025 |
| Shannon index | | 4.50 | 4.21 | 0.170 | 0.154 |
| Abundance (%) | |  |  |  |  |
| *f_Acidaminococcaceae* | | 2.28 | 3.00 | 0.254 | 0.397 |
| *f_Bacteroidaceae* | | 0.01 | 0.51 | 0.528 | 0.228 |
| *f_Bacteroidales* | | 12.5 | 12.6 | 0.134 | 0.943 |
| *f_Christensenellaceae* | | 1.58 | 0.69 | 0.151 | 0.129 |
| *f_Clostridiaceae* | | 4.15 | 0.13 | 0.316 | 0.020 |
| *f_Elusimicrobiaceae* | | 0.11 | 0.12 | 0.364 | 0.506 |
| *f_Erysipelotrichaceae* | | 0.39 | 0.71 | 0.128 | 0.194 |
| *f_Family_XIII* |  | 0.18 | 0.22 | 0.068 | 0.218 |
| *f_Fibrobacteraceae* | | 0.74 | 2.16 | 0.112 | 0.019 |
| *f_Lachnospiraceae* | | 5.65 | 15.20 | 0.134 | 0.027 |
| *f_Porphyromonadaceae* | | 0.05 | 0.24 | 0.089 | 0.003 |
| *f_Prevotellaceae* | | 50.1 | 45.7 | 0.057 | 0.638 |
| *f_Rhodospirillaceae* | | 0.82 | 0.12 | 0.102 | 0.001 |
| *f_Rikenellaceae* | | 8.54 | 4.91 | 0.107 | 0.061 |
| *f_Ruminococcaceae* | | 8.43 | 8.56 | 0.224 | 0.586 |
| *f_Spirochaetaceae* | | 0.55 | 2.42 | 0.277 | 0.130 |
| *f_Succinivibrionaceae* | | 0.25 | 0.52 | 0.125 | 0.088 |
| *f_Synergistaceae* | | 0.14 | 0.10 | 0.312 | 0.981 |
| *f_Veillonellaceae* | | 0.46 | 1.28 | 0.109 | 0.020 |
| Methanogens community | | |  |  |  |
| Concentration (log10 copies/ml) | | 6.32 | 7.65 | 1.213 | 0.314 |
| Richness |  | 20.5 | 14.5 | 1.683 | 0.025 |
| Shannon index | | 1.85 | 1.75 | 0.240 | 0.688 |
| Abundance (%) | |  |  |  |  |
| *f_Methanobacteriaceae* | | 35.8 | 55.2 | 33.30 | 0.043 |
| *f_Methanomassiliicoccaceae* | | 62.6 | 44.8 | 38.00 | 0.079 |
| *f_Methanomicrobiaceae* | | 1.61 | 0.00 | 4.620 | 0.158 |
| *g_Methanobrevibacter* | | 28.7 | 55.2 | 23.10 | 0.005 |
| *g_Methanosphaera* | | 7.12 | 0.00 | 15.88 | 0.089 |
| Protozoal community | |  |  |  |  |
| Concentration (log10 copies/ml) | | 6.60 | 8.70 | 0.493 | 0.005 |
| Richness |  | 25.0 | 27.0 | 2.890 | 0.527 |
| Shannon index | | 2.24 | 2.46 | 0.187 | 0.297 |
| Abundance (%) | |  |  |  |  |
| *g_Entodinium* |  | 48.8 | 49.3 | 0.102 | 0.863 |
| *g_Ophryoscolex* | | 3.19 | 9.02 | 0.737 | 0.126 |
| *g_Diplodinium* |  | 0.01 | 0.01 | 0.123 | 0.999 |
| *g_Polyplastron* | | 0.66 | 2.84 | 0.548 | 0.087 |
| *g_Enoploplastron* | | 0.83 | 0.00 | 0.101 | <0.001 |
| *g_Isotricha* |  | 18.6 | 22.7 | 0.215 | 0.969 |
| *g_Dasytricha* |  | 25.5 | 9.4 | 0.046 | <0.001 |
| *g_*Unclassified |  | 2.31 | 6.70 | 0.481 | 0.121 |
| Anaerobic fungal community | | |  |  |  |
| Concentration (log10 copies/ml) | | 6.76 | 7.55 | 0.125 | <0.001 |
| Richness |  | 12.0 | 15.0 | 2.380 | 0.276 |
| Shannon index | | 1.32 | 1.63 | 0.527 | 0.501 |
| Abundance (%) | |  |  |  |  |
| *g_Caecomyces* | | 63.6 | 9.22 | 0.655 | 0.064 |
| *g_Piromyces* |  | 0.00 | 50.0 | 0.006 | <0.001 |
| *g_Neocallimastigaceae* | | 36.4 | 40.8 | 0.107 | 0.621 |

^1^Treatments: Autoclaved rumen fluid (AUT), fresh rumen fluid from adult goats adapted to forage-rich (RFF) or concentrate-rich diet (RFC). s.e.d.; standard error of the difference and *P*-values for microbial taxa abundance were calculated based on the log10 transformed number of sequences.

**Supplementary Table S2.** Effect of the early-in-life rumen microbial inoculation and age on the rumen bacteria concentration, diversity and taxonomy.

| **Time** | **5 weeks** | | | | **7 weeks** | | | | **9 weeks** | | | |  | ***P-value*** | | |
| --- | --- | --- | --- | --- | --- | --- | --- | --- | --- | --- | --- | --- | --- | --- | --- | --- |
| **Inoculation^1^** | **CTL** | **AUT** | **RFF** | **RFC** | **CTL** | **AUT** | **RFF** | **RFC** | **CTL** | **AUT** | **RFF** | **RFC** | **s.e.d.** | **Inoc.** | **Time** | **IxT** |
| **Diversity** |  |  |  |  |  |  |  |  |  |  |  |  |  |  |  |  |
| Concentation (log10 copies/mg DM) | 10.7 | 10.9 | 10.8 | 10.7 | 10.8 | 10.7 | 10.7 | 10.4 | 10.7 | 10.7 | 10.7 | 10.4 | 0.11 | 0.004 | 0.003 | 0.543 |
| Richness | 151 | 281 | 421 | 350 | 206 | 388 | 510 | 453 | 145 | 294 | 379 | 326 | 48.09 | <0.001 | <0.001 | 0.900 |
| Shannon index | 2.83 | 3.10 | 4.17 | 3.61 | 2.99 | 3.78 | 4.37 | 4.16 | 2.57 | 3.19 | 3.73 | 3.42 | 0.240 | <0.001 | <0.001 | 0.599 |
| Simpson index | 0.84 | 0.83 | 0.95 | 0.91 | 0.87 | 0.91 | 0.95 | 0.94 | 0.80 | 0.85 | 0.90 | 0.87 | 0.030 | <0.001 | <0.001 | 0.673 |
| Evenness | 0.57 | 0.55 | 0.69 | 0.62 | 0.56 | 0.64 | 0.7 | 0.68 | 0.52 | 0.57 | 0.63 | 0.59 | 0.032 | <0.001 | <0.001 | 0.379 |
| Chao Index | 195^g^ | 435^def^ | 582^bcd^ | 758^b^ | 348^efg^ | 564^bcde^ | 985^a^ | 661^bc^ | 226^fg^ | 453^cde^ | 598^bcd^ | 545^bcde^ | 111.4 | <0.001 | 0.003 | 0.072 |
| Good´s coverage | 0.71^a^ | 0.63^bc^ | 0.65^ab^ | 0.54^d^ | 0.63^bc^ | 0.63^bc^ | 0.57^cd^ | 0.63^bc^ | 0.65^ab^ | 0.59^bcd^ | 0.60^bcd^ | 0.61^bcd^ | 0.036 | 0.015 | 0.439 | 0.014 |
| **Abundance (log10 sequences)** |  |  |  |  |  |  |  |  |  |  |  |  |  |  |  |  |
| *p_Actinobacteria, f_Coriobacteriaceae* | 0.97 | 0.66 | 0.82 | 0.64 | 1.02 | 0.82 | 0.63 | 0.7 | 0.26 | 0.37 | 0.42 | 0.46 | 0.196 | 0.642 | <0.001 | 0.243 |
| *g_Atopobium* | 0.75 | 0.66 | 0.69 | 0.41 | 0.91 | 0.72 | 0.45 | 0.38 | 0.08 | 0.22 | 0.19 | 0.13 | 0.202 | 0.152 | <0.001 | 0.361 |
| *g_Olsenella* | 0.44 | 0.04 | 0.22 | 0.34 | 0.24 | 0.19 | 0.29 | 0.41 | 0.2 | 0.17 | 0.23 | 0.35 | 0.162 | 0.242 | 0.818 | 0.603 |
| *p_Bacteroidetes* | 3.67 | 3.69 | 3.59 | 3.62 | 3.56 | 3.61 | 3.59 | 3.57 | 3.35 | 3.44 | 3.5 | 3.49 | 0.056 | 0.339 | <0.001 | 0.142 |
| *f_Bacteroidaceae, g_Bacteroides* | 2.15^a^ | 0.74^b^ | 0.09^c^ | 0.04^c^ | 0.48^bc^ | 0.33^bc^ | 0.04^c^ | 0.04^c^ | 0.04^c^ | 0.10^c^ | 0^c^ | 0.04^c^ | 0.183 | <0.001 | <0.001 | <0.001 |
| *f_Bacteroidaceae* | 3.35^a^ | 3.29^a^ | 2.89^ab^ | 2.82^abc^ | 2.80^abc^ | 3.19^a^ | 2.94^ab^ | 3.01^a^ | 1.69^d^ | 2.13^cd^ | 2.29^bcd^ | 2.05^d^ | 0.202 | 0.110 | <0.001 | 0.017 |
| *g_Phocaeicola* | 0.44^b^ | 1.24^a^ | 1.30^c^ | 0.91^c^ | 0.95^bc^ | 1.08^bc^ | 1.02^c^ | 1.11^c^ | 0.67^c^ | 0.67^c^ | 0.93^c^ | 0.75^c^ | 0.173 | 0.003 | 0.005 | 0.016 |
| *f_p-2534-18B5_gut_group* | 0.29^c^ | 1.37^abc^ | 0.85^bc^ | 2.12^ab^ | 2.57^a^ | 1.15^abc^ | 1.48^abc^ | 1.71^abc^ | 1.16^abc^ | 1.52^abc^ | 1.61^abc^ | 1.03^bc^ | 0.432 | 0.494 | 0.039 | <0.001 |
| *f_PeH15* | 0^c^ | 0.06^c^ | 1.54^a^ | 0.97^ab^ | 0^c^ | 0.13^bc^ | 0.73^bc^ | 0.56^bc^ | 0^c^ | 0.04^c^ | 0.16^bc^ | 0.04^c^ | 0.232 | <0.001 | <0.001 | <0.001 |
| *f_Porphyromonadaceae* | 0.81^a^ | 0.29^b^ | 0.11^b^ | 0.15^b^ | 0.16^b^ | 0.15^b^ | 0.13^b^ | 0.19^b^ | 0.11^b^ | 0.19^b^ | 0.28^b^ | 0.11^b^ | 0.134 | 0.058 | 0.016 | <0.001 |
| *f_Prevotellaceae* | 3.16 | 3.17 | 3.25 | 3.19 | 3.25 | 3.27 | 3.33 | 3.25 | 3.29 | 3.38 | 3.41 | 3.46 | 0.077 | 0.177 | <0.001 | 0.843 |
| *g_Alloprevotella* | 1.90 | 2.10 | 1.36 | 1.36 | 1.83 | 1.28 | 1.15 | 0.92 | 0.67 | 0.70 | 0.52 | 0.42 | 0.304 | 0.019 | <0.001 | 0.404 |
| *g_Prevotella* | 2.99 | 2.86 | 3.00 | 3.05 | 2.97 | 3.19 | 3.21 | 3.1 | 3.22 | 3.21 | 3.29 | 3.34 | 0.130 | 0.484 | <0.001 | 0.547 |
| *f_Rikenellaceae* | 1.62^d^ | 2.00^bcd^ | 2.83^a^ | 2.82^a^ | 2.19^abcd^ | 2.38^abcd^ | 2.69^ab^ | 2.53^abc^ | 1.72^d^ | 1.70^d^ | 1.95^bcd^ | 1.82^cd^ | 0.228 | <0.001 | <0.001 | 0.019 |
| *g_Alistipes* | 0.78^a^ | 0.11^bc^ | 0^c^ | 0^c^ | 0.41^b^ | 0.20^bc^ | 0.06^c^ | 0^c^ | 0^c^ | 0^c^ | 0^c^ | 0^c^ | 0.087 | <0.001 | <0.001 | <0.001 |
| *g_SP3-e08* | 0^a^ | 1.23^bc^ | 1.69^c^ | 1.66^c^ | 0.45^b^ | 1.83^bc^ | 1.17^c^ | 1.23^c^ | 0^c^ | 0.55^c^ | 0.47^c^ | 0.26^c^ | 0.337 | <0.001 | <0.001 | 0.021 |
| *p_Chloroflexi, f_Anaerolineaceae* | 0^d^ | 0.86^ab^ | 1.13^a^ | 0.95^a^ | 0.08^d^ | 0.69^abc^ | 0.78^ab^ | 0.77^ab^ | 0^d^ | 0^d^ | 0.26^bcd^ | 0.16^cd^ | 0.169 | <0.001 | <0.001 | 0.003 |
| *p_Cyanobacteria, f_Gastranaerophilales* | 0 | 0 | 0.23 | 0.10 | 0 | 0.08 | 0.34 | 0.44 | 0 | 0 | 0.29 | 0.06 | 0.138 | <0.001 | 0.126 | 0.554 |
| *p_Elusimicrobia* | 0 | 1.1 | 0.87 | 0.98 | 0.34 | 1.06 | 1.01 | 1.04 | 0 | 0.19 | 0.39 | 0.06 | 0.205 | <0.001 | <0.001 | 0.063 |
| *f_Clostridiaceae* | 0^d^ | 0.92^a^ | 0.65^abc^ | 0.55^abcd^ | 0.21^bcd^ | 0.70^ab^ | 0.53^abcd^ | 0.28^abcd^ | 0^d^ | 0^d^ | 0^cd^ | 0^d^ | 0.184 | <0.001 | <0.001 | 0.05 |
| *f_Elusimicrobiaceae, g_Elusimicrobium* | 0^b^ | 0^b^ | 0.39^ab^ | 0.61^a^ | 0^b^ | 0.34^ab^ | 0.61^a^ | 0.46^ab^ | 0^b^ | 0.19^ab^ | 0.33^ab^ | 0.04^b^ | 0.158 | <0.001 | 0.028 | 0.027 |
| *p_Fibrobacteres, g_Fibrobacter* | 0 | 0.75 | 1.11 | 0.71 | 0.44 | 0.95 | 1.16 | 0.86 | 0.36 | 0.48 | 1.53 | 0.92 | 0.212 | <0.001 | 0.108 | 0.106 |
| *p_Firmicutes* | 3.05 | 2.82 | 3.18 | 3.01 | 3.07 | 2.92 | 3.11 | 3.16 | 2.99 | 3.02 | 3.08 | 3.15 | 0.081 | <0.001 | 0.468 | 0.135 |
| *f_Acidaminococcaceae* | 1.48 | 1.32 | 1.73 | 1.63 | 1.11 | 1.31 | 1.70 | 1.67 | 1.42 | 1.23 | 1.85 | 1.55 | 0.179 | <0.001 | 0.56 | 0.553 |
| *g_Acidaminococcus* | 0.36 | 0.12 | 0 | 0.22 | 0.04 | 0.23 | 0.13 | 0.07 | 0.55 | 0.51 | 0.36 | 0.37 | 0.171 | 0.413 | <0.001 | 0.513 |
| *g_Phascolarctobacterium* | 1.00^a^ | 0.06^b^ | 0^b^ | 0^b^ | 0.21^b^ | 0.10^b^ | 0.04^b^ | 0^b^ | 0^b^ | 0^b^ | 0^b^ | 0^b^ | 0.110 | <0.001 | <0.001 | <0.001 |
| *g_Succiniclasticum* | 0.61 | 1.31 | 1.73 | 1.63 | 1.04 | 1.28 | 1.69 | 1.66 | 1.28 | 1.11 | 1.84 | 1.53 | 0.207 | <0.001 | 0.489 | 0.127 |
| *f_Christensenellaceae* | 2.11 | 2.03 | 2.50 | 2.07 | 1.72 | 1.81 | 2.01 | 1.91 | 0.41 | 0.68 | 0.89 | 0.92 | 0.237 | 0.137 | <0.001 | 0.613 |
| *f_Clostridiales* | 1.4 | 1.48 | 1.56 | 1.2 | 1.22 | 1.25 | 1.51 | 1.36 | 0.16 | 0.43 | 0.69 | 0.30 | 0.224 | 0.263 | <0.001 | 0.607 |
| *f_Defluviitaleaceae* | 0.04^c^ | 0.06^c^ | 0.36^abc^ | 0.51^ab^ | 0.06^c^ | 0.10^bc^ | 0.70^a^ | 0.66^a^ | 0.04^c^ | 0.11^bc^ | 0.16^bc^ | 0.21^bc^ | 0.123 | <0.001 | <0.001 | 0.007 |
| *f_Erysipelotrichaceae* | 0.66 | 1.11 | 1.14 | 0.93 | 0.58 | 1.08 | 0.86 | 0.98 | 0.4 | 0.5 | 0.99 | 0.71 | 0.195 | 0.019 | 0.002 | 0.152 |
| *g_Catenisphaera* | 0 | 0.46 | 0.08 | 0.19 | 0 | 0.22 | 0 | 0.06 | 0 | 0 | 0 | 0.11 | 0.131 | 0.096 | 0.028 | 0.131 |
| *g_Sharpea* | 0.60 | 0.11 | 0 | 0.06 | 0.52 | 0.07 | 0 | 0 | 0.35 | 0.14 | 0.15 | 0.04 | 0.158 | <0.001 | 0.824 | 0.633 |
| *g_Eubacterium* | 1.11 | 1.61 | 1.64 | 1.57 | 1.08 | 1.57 | 1.52 | 1.51 | 1.13 | 1.64 | 1.76 | 1.52 | 0.205 | 0.005 | 0.565 | 0.984 |
| *f_Family_XIII* | 0.63^efg^ | 1.25^bcde^ | 1.95^a^ | 1.51^abc^ | 1.40^abc^ | 1.37^abcd^ | 1.78^ab^ | 1.65^ab^ | 0.15^g^ | 0.56^fg^ | 0.93^cdef^ | 0.70^defg^ | 0.19 | <0.001 | <0.001 | 0.022 |
| *g_Anaerovorax* | 0.08^e^ | 0.70^bcd^ | 1.38^a^ | 0.94^abc^ | 0.75^bc^ | 0.78^bc^ | 1.15^ab^ | 1.10^ab^ | 0.04^e^ | 0.20^de^ | 0.43^cde^ | 0.17^de^ | 0.151 | <0.001 | <0.001 | <0.001 |
| *g_Mogibacterium* | 0.21^cd^ | 0.34^cd^ | 1.15^a^ | 0.99^ab^ | 0.29^cd^ | 0.77^abc^ | 1.09^a^ | 0.98^ab^ | 0.08^d^ | 0.22^d^ | 0.39^bcd^ | 0.36^cd^ | 0.171 | <0.001 | <0.001 | 0.007 |
| *f_Lachnospiraceae* | 2.41 | 2.22 | 2.55 | 2.31 | 2.75 | 2.62 | 2.55 | 2.6 | 2.83 | 2.74 | 2.82 | 2.90 | 0.145 | 0.420 | <0.001 | 0.407 |
| *g_Acetitomaculum* | 0.45 | 0.70 | 1.28 | 1.21 | 0.63 | 1.11 | 1.20 | 1.13 | 0.71 | 0.67 | 0.83 | 1.00 | 0.244 | 0.008 | 0.180 | 0.301 |
| *g_Anaerosporobacter* | 0.48^ab^ | 0.41^ab^ | 0.38^b^ | 0^b^ | 0.52^ab^ | 1.02^a^ | 0.19^b^ | 0.08^b^ | 0^b^ | 0.15^b^ | 0.06^b^ | 0.06^b^ | 0.178 | <0.001 | <0.001 | 0.011 |
| *g_Butyrivibrio* | 1.30^b^ | 1.47^ab^ | 1.33^ab^ | 1.16^b^ | 2.01^a^ | 1.53^ab^ | 1.25^b^ | 1.14^b^ | 0.86^b^ | 1.14^b^ | 1.35^ab^ | 1.27^b^ | 0.194 | 0.238 | 0.008 | <0.001 |
| *g_Coprococcus* | 1.00 | 0.64 | 0.51 | 0.39 | 0.60 | 0.89 | 0.43 | 0.39 | 0.15 | 0.42 | 0.18 | 0.15 | 0.245 | 0.032 | 0.005 | 0.515 |
| *g_Lachnoclostridium* | 0.04 | 0.50 | 1.02 | 0.61 | 0.17 | 0.64 | 1.08 | 1.08 | 0.72 | 1.15 | 1.41 | 1.72 | 0.223 | <0.001 | <0.001 | 0.369 |
| *g_Lachnospira* | 0.58^a^ | 0.24^bcde^ | 0^e^ | 0.04^cde^ | 0.04^cde^ | 0.37^abc^ | 0.06^cde^ | 0^de^ | 0.44^ab^ | 0.22^bcde^ | 0.33^abcd^ | 0.21^bcde^ | 0.169 | 0.077 | 0.085 | 0.03 |
| *g_Lachnospiraceae* | 1.80 | 1.71 | 2.23 | 1.87 | 2.39 | 2.26 | 2.22 | 2.18 | 2.1 | 2.26 | 2.39 | 2.54 | 0.232 | 0.622 | <0.001 | 0.151 |
| *g_Marvinbryantia* | 0 | 0.16 | 0.34 | 0.18 | 0.08 | 0.23 | 0.17 | 0.36 | 0.14 | 0.04 | 0.26 | 0.32 | 0.117 | 0.06 | 0.781 | 0.123 |
| *g_Moryella* | 0.43 | 0.79 | 1.21 | 1.14 | 0.68 | 1.01 | 1.56 | 1.77 | 0.53 | 1.13 | 1.38 | 1.62 | 0.188 | <0.001 | <0.001 | 0.552 |
| *g_Oribacterium* | 0.46 | 0.59 | 0.72 | 0.58 | 0.57 | 0.8 | 0.89 | 0.81 | 0.83 | 1.25 | 1.20 | 1.59 | 0.233 | 0.006 | <0.001 | 0.63 |
| *g_Pseudobutyrivibrio* | 0.60 | 0.80 | 0.83 | 0.50 | 0.78 | 0.98 | 0.79 | 0.89 | 1.01 | 1.04 | 0.95 | 1.02 | 0.234 | 0.597 | 0.037 | 0.896 |
| *g_Roseburia* | 0.77 | 0.52 | 0.53 | 0.58 | 0.74 | 1.44 | 1.17 | 1.00 | 1.80 | 1.73 | 1.52 | 1.48 | 0.267 | 0.647 | <0.001 | 0.18 |
| *g_Syntrophococcus* | 0.27 | 0.13 | 0.34 | 0.19 | 0.16 | 0.35 | 0.13 | 0.34 | 0.28 | 0.15 | 0.29 | 0.33 | 0.122 | 0.792 | 0.893 | 0.136 |
| *g_Tyzzerella* | 0.55 | 0.39 | 0.20 | 0.06 | 0.30 | 0.25 | 0.13 | 0.07 | 0 | 0 | 0.12 | 0 | 0.14 | 0.032 | 0.001 | 0.192 |
| *f_Peptococcaceae* | 0.36 | 0.56 | 0.62 | 0.33 | 0.25 | 0.19 | 0.34 | 0.34 | 0 | 0 | 0 | 0 | 0.152 | 0.684 | <0.001 | 0.49 |
| *f_Ruminococcaceae* | 2.57 | 2.38 | 2.59 | 2.45 | 2.4 | 2.31 | 2.70 | 2.55 | 2.02 | 1.82 | 2.35 | 2.07 | 0.152 | 0.037 | <0.001 | 0.457 |
| *g_Anaerotruncus* | 0.14 | 0 | 0.51 | 0.26 | 0.25 | 0.15 | 0.55 | 0.47 | 0 | 0.10 | 0.15 | 0.08 | 0.109 | <0.001 | <0.001 | 0.127 |
| *g_Oscillibacter* | 0.69 | 0.52 | 0.10 | 0.08 | 0.59 | 0.44 | 0.13 | 0.21 | 0.25 | 0.16 | 0.15 | 0.04 | 0.119 | <0.001 | 0.002 | 0.06 |
| *g_Papillibacter* | 0.17 | 0.07 | 0.76 | 0.45 | 0.29 | 0.47 | 0.55 | 0.45 | 0 | 0.04 | 0.23 | 0 | 0.148 | 0.003 | <0.001 | 0.083 |
| *g_Ruminiclostridium* | 0.80^a^ | 0.75^a^ | 0.5^abc^ | 0.31^abc^ | 0.67^a^ | 0.70^a^ | 0.63^ab^ | 0.64^ab^ | 0.04^c^ | 0.07^bc^ | 0.23^abc^ | 0.41^abc^ | 0.171 | 0.871 | <0.001 | 0.033 |
| *g_Ruminococcus* | 1.63^ab^ | 1.04^cd^ | 1.41^abc^ | 1.12^cd^ | 1.25^abcd^ | 1.40^abc^ | 1.65^ab^ | 1.30^abcd^ | 0.87^d^ | 1.18^bcd^ | 1.69^a^ | 1.60^ab^ | 0.243 | 0.090 | 0.675 | 0.011 |
| *g_Saccharofermentans* | 0 | 0.06 | 0.27 | 0.08 | 0 | 0.1 | 0.48 | 0.43 | 0 | 0.06 | 0.50 | 0.63 | 0.144 | <0.001 | 0.022 | 0.133 |
| *g_Streptococcus* | 0.22^a^ | 0^b^ | 0.08^ab^ | 0^b^ | 0^b^ | 0.04^ab^ | 0.04^ab^ | 0^b^ | 0^b^ | 0^b^ | 0^b^ | 0^b^ | 0.057 | 0.114 | 0.037 | 0.038 |
| *f_Veillonellaceae* | 0.73^f^ | 0.70^f^ | 1.16^cdef^ | 1.02^def^ | 0.92^ef^ | 1.28^cdef^ | 1.6^cde^ | 1.72^abc^ | 1.62^bcd^ | 2.42^a^ | 1.81^abc^ | 2.34^ab^ | 0.205 | 0.002 | <0.001 | 0.002 |
| *g_Anaerovibrio* | 0.43^c^ | 0.16^c^ | 0.16^c^ | 0.19^c^ | 0.34^c^ | 0.47^bc^ | 0.47^bc^ | 0.49^bc^ | 0.62^bc^ | 1.26^a^ | 0.58^bc^ | 1.10^ab^ | 0.179 | 0.12 | <0.001 | 0.008 |
| *g_Megasphaera* | 0.25 | 0.08 | 0 | 0.11 | 0.14 | 0.07 | 0.04 | 0.07 | 0.51 | 0.32 | 0.31 | 0.28 | 0.129 | 0.201 | <0.001 | 0.938 |
| *g_Quinella* | 0^d^ | 0^d^ | 0.50^bcd^ | 0.16^cd^ | 0^d^ | 0.16^cd^ | 0.97^abc^ | 1.15^ab^ | 0^d^ | 1.15^ab^ | 0.73^abcd^ | 1.45^a^ | 0.247 | <0.001 | <0.001 | <0.001 |
| *g_Selenomonas* | 0^e^ | 0.10^de^ | 0.35^de^ | 0.43^de^ | 0.37^de^ | 0.51^cde^ | 0.67^cde^ | 0.89^bcd^ | 0.79^bcde^ | 1.96^a^ | 1.33^abc^ | 1.62^ab^ | 0.243 | 0.004 | <0.001 | 0.033 |
| *p_Proteobacteria* | 1.82 | 2.40 | 1.95 | 2.36 | 2.57 | 2.63 | 2.50 | 2.15 | 3.41 | 3.02 | 2.91 | 2.78 | 0.285 | 0.45 | <0.001 | 0.065 |
| *f_Campylobacteraceae, g_Campylobacter* | 0.32 | 0.04 | 0.19 | 0.08 | 0 | 0 | 0.10 | 0.09 | 0.08 | 0.04 | 0.04 | 0 | 0.112 | 0.333 | 0.069 | 0.423 |
| *f_Comamonadaceae, g_Comamonas* | 0.75^a^ | 0.04^b^ | 0^b^ | 0^b^ | 0.23^b^ | 0.18^b^ | 0.07^b^ | 0.04^b^ | 0^b^ | 0.04^b^ | 0^b^ | 0.04^b^ | 0.125 | 0.004 | 0.012 | <0.001 |
| *f_Neisseriaceae* | 0.74 | 0.96 | 0.63 | 0.55 | 0.38 | 0.33 | 0.42 | 0.32 | 0.04 | 0 | 0 | 0.06 | 0.146 | 0.679 | <0.001 | 0.189 |
| *f_Pasteurellaceae* | 0.80^a^ | 0.35^bcd^ | 0.64^ab^ | 0.26^bcd^ | 0.34^bcd^ | 0.22^bcd^ | 0.22^cd^ | 0.46^abc^ | 0^d^ | 0.06^cd^ | 0.04^cd^ | 0^d^ | 0.12 | 0.006 | <0.001 | 0.001 |
| *f_Pasteurellaceae, g_Bibersteinia* | 0.62^a^ | 0.25^abc^ | 0.39^ab^ | 0.23^bc^ | 0.27^bc^ | 0.16^bc^ | 0.19^bc^ | 0.34^abc^ | 0^c^ | 0.06^bc^ | 0^c^ | 0^c^ | 0.105 | 0.223 | <0.001 | 0.012 |
| *f_Rhodocyclaceae* | 0^d^ | 0.62^ab^ | 0.72^a^ | 0.36^abcd^ | 0.11^cd^ | 0.29^abcd^ | 0.21^bcd^ | 0.52^abc^ | 0^d^ | 0.04^cd^ | 0^d^ | 0^d^ | 0.138 | 0.006 | <0.001 | 0.001 |
| *f_Rhodospirillaceae* | 0.48 | 0.56 | 1 | 0.68 | 0.35 | 0.5 | 0.94 | 0.9 | 0.16 | 0.74 | 1.27 | 0.34 | 0.250 | 0.001 | 0.890 | 0.127 |
| *f_Succinivibrionaceae* | 0.78^d^ | 2.21^abc^ | 1.59^cd^ | 2.11^abc^ | 2.43^abc^ | 2.59^abc^ | 2.41^abc^ | 1.91^bcd^ | 3.41^a^ | 3.01^ab^ | 2.81^abc^ | 2.77^abc^ | 0.372 | 0.349 | <0.001 | 0.002 |
| *g_Anaerobiospirillum* | 0 | 0 | 0.27 | 0.04 | 0 | 0.12 | 0.19 | 0 | 0 | 0.28 | 0.5 | 0.32 | 0.115 | <0.001 | 0.002 | 0.403 |
| *g_Ruminobacter* | 0.08^c^ | 2.07^a^ | 0.97^abc^ | 1.5^ab^ | 0.78^bc^ | 1.17^abc^ | 1.46^ab^ | 0.74^bc^ | 0.29^bc^ | 0.81^bc^ | 0.31^bc^ | 0.70^bc^ | 0.344 | 0.001 | <0.001 | 0.003 |
| *g_Succinimonas* | 0 | 0.11 | 0.5 | 0.35 | 0 | 0.36 | 0.27 | 0.28 | 0 | 0.04 | 0.25 | 0 | 0.215 | 0.055 | 0.244 | 0.684 |
| *g_Succinivibrio* | 0.71 | 0.88 | 0.79 | 1.23 | 2.29 | 1.92 | 1.56 | 1.45 | 3.40 | 2.5 | 2.62 | 2.66 | 0.483 | 0.475 | <0.001 | 0.345 |
| *p_Spirochaetae, f_Spirochaetaceae* | 0.95^b^ | 2.04^a^ | 1.68^ab^ | 1.72^ab^ | 1.80^ab^ | 2.02^a^ | 1.84^ab^ | 1.67^ab^ | 1.42^ab^ | 2.09^a^ | 2.08^a^ | 1.25^ab^ | 0.277 | 0.005 | 0.206 | 0.046 |
| *g_Sphaerochaeta* | 0.82 | 1.69 | 0.82 | 1.26 | 0.63 | 1.31 | 0.99 | 1.12 | 0.2 | 0.63 | 0.66 | 0.21 | 0.278 | 0.007 | <0.001 | 0.249 |
| *g_Treponema* | 0.16^b^ | 1.72^a^ | 1.49^a^ | 1.24^a^ | 1.60^a^ | 1.85^a^ | 1.63^a^ | 1.19^a^ | 1.41^a^ | 2.06^a^ | 2.04^a^ | 1.22^a^ | 0.291 | <0.001 | <0.001 | 0.005 |
| *p_Synergistetes, f_Synergistaceae* | 1.48^ab^ | 1.36^ab^ | 1.10^abc^ | 0.82^abcd^ | 1.07^abcd^ | 1.07^abcd^ | 1.32^abc^ | 1.59^a^ | 0.25^d^ | 0.49^cd^ | 0.68^bcd^ | 1.08^abcd^ | 0.240 | 0.411 | <0.001 | 0.001 |
| *g_Fretibacterium* | 0.04^c^ | 0.04^c^ | 0.65^abc^ | 0.34^bc^ | 0.14^c^ | 0.42^bc^ | 1.17^ab^ | 1.47^a^ | 0.08^c^ | 0.36^bc^ | 0.42^bc^ | 0.90^abc^ | 0.256 | <0.001 | <0.001 | 0.032 |
| *g_Pyramidobacter* | 1.47^a^ | 1.03^ab^ | 0.76^bcd^ | 0.68^bcde^ | 0.80^bc^ | 0.76^bcd^ | 0.59^bcde^ | 0.68^bcde^ | 0.19^e^ | 0.25^de^ | 0.30^cde^ | 0.37^cde^ | 0.151 | 0.007 | <0.001 | 0.003 |
| *g_Synergistes* | 0^b^ | 0.95^a^ | 0.18^b^ | 0.08^b^ | 0.54^ab^ | 0.34^b^ | 0.10^b^ | 0.10^b^ | 0^b^ | 0^b^ | 0^b^ | 0.08^b^ | 0.163 | 0.005 | 0.002 | <0.001 |
| *p_Tenericutes* | 0.37 | 0.77 | 1.27 | 1.16 | 0.29 | 0.66 | 1.07 | 1.09 | 0.29 | 0.17 | 0.73 | 0.42 | 0.19 | <0.001 | <0.001 | 0.132 |
| *f_Erysipelotrichaceae* | 0.66 | 1.11 | 1.14 | 0.93 | 0.58 | 1.08 | 0.86 | 0.98 | 0.40 | 0.5 | 0.99 | 0.71 | 0.195 | 0.019 | 0.002 | 0.152 |
| *f_Mollicutes_RF9_uncultured* | 0.14 | 0.15 | 0.53 | 0.39 | 0.10 | 0.14 | 0.44 | 0.47 | 0.13 | 0.13 | 0.31 | 0.04 | 0.141 | 0.002 | 0.077 | 0.28 |
| *f_NB1-n_uncultured* | 0 | 0.13 | 0.15 | 0.54 | 0 | 0.21 | 0.37 | 0.29 | 0 | 0 | 0.11 | 0.04 | 0.139 | 0.01 | 0.018 | 0.092 |
| *p_Minor phyla* | 0.22^cde^ | 0.58^bcd^ | 1.44^a^ | 0.97^ab^ | 0.22^cde^ | 0.34^cde^ | 0.76^bc^ | 0.97^ab^ | 0^e^ | 0.13^de^ | 0.21^de^ | 0.22^cde^ | 0.158 | <0.001 | <0.001 | <0.001 |
| *f_Oligosphaeraceae* | 0^c^ | 0.26^bc^ | 0.86^a^ | 0.50^ab^ | 0.04^c^ | 0.21^bc^ | 0.30^bc^ | 0.36^bc^ | 0^c^ | 0^c^ | 0.06^c^ | 0^c^ | 0.105 | <0.001 | <0.001 | <0.001 |

^1^Goat kids inoculated (I) with fresh rumen fluid from adult goats adapted to forage-rich (RFF) or concentrate-rich diets (RFC), autoclaved rumen fluid (AUT) or absence of inoculation as control (CTL) and sampled at different times. ^a-f^ Means within a row with different superscript differ (*P*<0.05) based on the Bonferroni test.

**Supplementary Table S3.** Spearman’s correlations between the bacterial taxa and the rumen function and animal performance.

| Correlations^1^ | Milk | Concentrate | Forage | DMI | pH | Ammonia | VFA | OBCVFA | Acetate | Propionate | Butyrate | Methanogens | Methanogens-R | Protozoa | Protozoal-R | Fungi | Fungal-R | BHB | Glucose | ADG | ADG-pw |
| --- | --- | --- | --- | --- | --- | --- | --- | --- | --- | --- | --- | --- | --- | --- | --- | --- | --- | --- | --- | --- | --- |
| Concentation |  | -0.32 |  |  |  |  |  |  |  |  |  |  |  |  |  |  |  |  |  |  |  |
| Richness |  | 0.45 | 0.51 | 0.46 |  |  |  |  |  |  |  | 0.31 | 0.55 | 0.55 |  |  |  |  |  |  |  |
| *p_Actinobacteria, f_Coriobacteriaceae* | -0.42 |  |  |  |  |  |  |  |  |  |  |  |  |  | -0.37 |  | -0.38 |  |  | -0.38 |  |
| *g_Atopobium* | -0.37 |  |  |  |  |  |  |  |  |  |  |  |  |  |  |  |  |  |  | -0.37 | -0.38 |
| *p_Bacteroidetes* |  | -0.39 | -0.40 | -0.50 |  |  |  |  |  |  | -0.34 |  |  |  |  |  |  | -0.33 | 0.36 |  | -0.46 |
| *f_Bacteroidaceae, g_Bacteroides* |  | -0.39 | -0.68 | -0.44 |  |  |  | 0.32 |  |  |  | -0.37 | -0.39 | -0.53 | -0.52 |  |  |  |  |  | -0.50 |
| *f_Bacteroidaceae* |  |  | -0.38 | -0.34 |  |  | -0.38 | 0.35 |  |  |  |  |  |  |  |  |  |  |  |  |  |
| *g_Phocaeicola* |  |  |  |  |  | 0.32 |  |  |  |  |  |  |  |  |  |  |  |  |  |  |  |
| *f_p-2534-18B5_gut_group* | 0.39 | 0.42 |  | 0.44 |  |  |  |  |  |  |  |  |  |  |  |  | 0.43 | 0.35 |  |  |  |
| *f_PeH15* |  |  | 0.44 |  | 0.34 |  |  |  |  |  |  |  |  | 0.54 | 0.63 |  |  |  |  |  |  |
| *f_Prevotellaceae* |  | 0.39 |  |  |  |  | 0.49 |  | -0.33 | 0.32 | 0.32 |  |  |  |  |  | -0.36 |  |  |  |  |
| *g_Alloprevotella* |  | -0.42 | -0.42 | -0.45 |  |  |  |  |  |  | -0.43 |  | -0.38 | -0.46 |  |  |  |  |  |  | -0.53 |
| *g_Prevotella* |  | 0.39 |  |  |  |  | 0.63 |  | -0.55 | 0.38 | 0.59 |  |  |  |  |  |  |  |  |  |  |
| *f_Rikenellaceae* |  |  | 0.45 |  |  |  |  |  | 0.35 |  |  |  |  | 0.46 | 0.58 |  |  |  |  |  |  |
| *g_Alistipes* |  |  | -0.54 |  |  |  |  |  |  |  |  | -0.38 | -0.41 | -0.61 | -0.65 |  |  |  |  |  |  |
| *g_SP3-e08* |  |  |  |  |  |  | -0.32 |  | 0.39 |  |  |  | 0.35 | 0.42 | 0.52 |  |  |  |  |  |  |
| *p_Chloroflexi, f_Anaerolineaceae* |  |  | 0.37 |  | 0.35 |  |  |  | 0.38 | -0.43 |  | 0.46 | 0.49 | 0.62 | 0.56 |  |  |  | -0.34 |  |  |
| *p_Cyanobacteria, f_Gastranaerophilales* |  | 0.49 | 0.53 | 0.49 |  |  |  | -0.37 |  |  |  |  |  | 0.52 | 0.73 |  |  |  |  |  |  |
| *p_Elusimicrobia* |  |  |  |  |  |  |  |  |  |  |  |  | 0.38 | 0.32 | 0.36 |  |  |  |  |  |  |
| *f_Elusimicrobiaceae, g_Elusimicrobium* |  |  | 0.41 |  |  |  |  |  |  |  |  |  | 0.46 | 0.59 | 0.55 |  |  |  |  |  |  |
| *p_Fibrobacteres, g_Fibrobacter* |  |  | 0.35 |  |  |  |  |  |  |  |  |  | 0.38 | 0.32 | 0.43 |  |  |  |  |  |  |
| *p_Firmicutes* |  |  | 0.48 |  |  |  |  |  |  |  |  |  |  |  |  | 0.37 |  |  | -0.49 |  | 0.39 |
| *f_Acidaminococcaceae* |  |  |  |  |  |  | 0.45 |  |  |  |  |  | 0.34 | 0.38 | 0.57 |  |  |  |  |  |  |
| *g_Acidaminococcus* |  |  |  |  |  |  |  |  |  |  |  |  |  |  |  |  | 0.34 |  |  | 0.35 |  |
| *g_Phascolarctobacterium* |  |  | -0.40 |  |  |  |  | 0.32 |  |  |  | -0.45 | -0.33 | -0.37 | -0.47 |  |  |  |  |  | -0.31 |
| *g_Succiniclasticum* |  |  | 0.41 |  |  |  | 0.43 | -0.37 |  |  |  |  | 0.35 | 0.46 | 0.65 |  |  |  |  |  |  |
| *f_Christensenellaceae* | -0.43 |  |  |  |  |  |  |  | 0.42 | -0.42 | -0.33 |  |  |  |  |  |  |  | -0.36 | -0.39 |  |
| *f_Clostridiaceae* | -0.39 |  |  |  |  |  |  |  | 0.37 | -0.37 |  |  |  |  |  |  |  |  |  | -0.44 |  |
| *f_Defluviitaleaceae* |  |  | 0.38 |  |  |  |  |  |  |  |  |  | 0.36 | 0.64 | 0.45 |  | -0.38 |  |  |  |  |
| *f_Erysipelotrichaceae* |  |  |  |  |  |  |  |  |  |  |  |  |  |  |  |  |  |  |  |  |  |
| *g_Sharpea* |  |  | -0.52 |  |  |  |  |  |  |  |  |  | -0.52 | -0.56 | -0.60 |  |  |  | 0.41 |  |  |
| *g_Eubacterium* |  |  |  |  |  |  |  |  |  |  |  |  | 0.34 |  | 0.57 |  |  |  |  |  |  |
| *f_Family_XIII* |  |  | 0.52 |  |  |  |  |  |  | -0.33 |  | 0.46 |  | 0.53 | 0.38 |  | -0.38 |  | -0.32 |  |  |
| *g_Anaerovorax* |  | 0.34 | 0.55 | 0.32 |  |  |  |  |  |  |  | 0.38 |  | 0.59 | 0.68 |  |  |  |  |  |  |
| *g_Mogibacterium* | -0.34 |  | 0.37 |  |  |  |  |  | 0.39 | -0.39 |  | 0.39 | 0.39 | 0.56 | 0.37 |  | -0.43 |  | -0.37 |  |  |
| *f_Lachnospiraceae* |  |  |  |  |  |  |  |  | -0.40 |  | 0.36 |  |  |  |  |  |  |  |  |  |  |
| *g_Acetitomaculum* |  |  | 0.36 |  |  |  |  |  |  |  |  | 0.36 | 0.43 | 0.47 |  |  |  |  |  |  |  |
| *g_Anaerosporobacter* |  |  |  |  |  |  |  |  | -0.39 | 0.31 |  |  |  | -0.31 |  |  |  |  |  |  |  |
| *g_Butyrivibrio* |  |  |  |  |  |  |  |  |  |  |  |  |  | -0.57 | -0.45 |  |  |  |  |  | -0.36 |
| *g_Coprococcus* |  |  |  |  |  |  |  |  |  |  |  |  |  |  |  |  |  |  |  |  | -0.44 |
| *g_Lachnoclostridium* |  |  | 0.41 |  |  |  |  |  |  |  | 0.32 | 0.45 | 0.56 | 0.53 | 0.68 |  |  |  |  |  |  |
| *g_Lachnospira* |  |  | -0.37 |  |  |  |  | 0.31 |  |  |  |  |  | -0.33 |  |  | 0.36 |  |  |  |  |
| *g_Marvinbryantia* |  |  |  |  |  |  |  |  |  |  |  |  | 0.32 |  |  |  | -0.37 |  |  |  |  |
| *g_Moryella* |  | 0.53 | 0.55 | 0.53 |  |  |  | -0.43 |  |  |  |  | 0.36 | 0.47 | 0.65 |  |  | 0.31 |  |  | 0.40 |
| *g_Oribacterium* |  |  | 0.40 | 0.31 |  |  |  |  |  |  |  |  |  |  | 0.46 |  |  |  |  |  |  |
| *g_Pseudobutyrivibrio* |  |  |  |  |  |  |  |  |  |  |  |  | 0.37 |  |  |  |  |  | -0.33 |  |  |
| *g_Roseburia* | 0.42 |  |  |  |  |  |  |  | -0.57 | 0.38 | 0.53 |  |  |  |  |  |  |  |  | 0.45 |  |
| *g_Syntrophococcus* |  |  |  |  |  |  |  | 0.44 |  |  |  |  |  |  |  |  |  |  |  |  |  |
| *g_Tyzzerella* |  | -0.39 |  | -0.39 |  |  |  | 0.34 |  |  |  |  |  |  | -0.35 |  |  |  |  |  | -0.33 |
| *f_Peptococcaceae* |  |  |  |  |  |  | -0.50 |  | 0.47 | -0.38 | -0.50 | 0.34 |  |  |  |  |  |  |  |  | -0.49 |
| *f_Ruminococcaceae* |  |  | 0.36 |  |  |  |  |  | 0.39 |  |  |  |  | 0.34 | 0.34 |  | -0.32 |  |  | -0.33 |  |
| *g_Anaerotruncus* |  | 0.36 | 0.42 | 0.37 |  |  |  |  |  |  |  |  |  |  | 0.33 |  |  |  |  |  |  |
| *g_Oscillibacter* |  |  | -0.44 |  | -0.33 |  |  |  |  |  |  |  | -0.36 | -0.57 | -0.39 |  | 0.43 |  | 0.32 |  |  |
| *g_Papillibacter* |  |  | 0.44 |  |  |  |  |  | 0.39 |  |  | 0.43 |  | 0.32 | 0.34 |  |  |  |  |  |  |
| *g_Ruminiclostridium* |  |  |  |  |  |  |  |  |  |  |  |  |  |  |  |  |  |  |  |  | -0.35 |
| *g_Ruminococcus* |  |  |  |  |  |  | 0.38 |  |  |  |  |  |  |  |  |  | -0.35 | 0.35 |  |  |  |
| *g_Saccharofermentans* |  | 0.39 | 0.35 | 0.40 |  |  |  |  |  |  |  |  |  | 0.46 | 0.45 |  |  | 0.36 |  |  | 0.58 |
| *g_Streptococcus* | -0.32 | -0.41 |  | -0.33 |  |  |  |  |  |  |  |  |  |  |  |  |  |  |  |  |  |
| *f_Veillonellaceae* |  | 0.54 | 0.47 | 0.55 |  |  | 0.35 |  | -0.44 |  | 0.58 |  | 0.44 | 0.34 | 0.53 |  |  | 0.37 |  |  | 0.47 |
| *g_Anaerovibrio* |  |  |  |  |  | 0.37 |  |  | -0.50 |  | 0.48 |  |  |  |  |  |  |  |  | 0.33 |  |
| *g_Megasphaera* |  |  | -0.32 |  |  |  |  |  |  |  |  | -0.33 |  | -0.32 |  |  | 0.32 |  |  |  |  |
| *g_Quinella* |  |  | 0.51 | 0.37 |  |  |  |  |  |  |  |  | 0.52 | 0.54 | 0.66 |  |  | 0.34 |  |  | 0.31 |
| *g_Selenomonas* | 0.32 | 0.54 | 0.53 | 0.58 |  |  |  |  | -0.37 |  | 0.45 |  | 0.36 |  | 0.45 |  |  | 0.38 |  |  | 0.41 |
| *p_Proteobacteria* | 0.53 | 0.34 |  | 0.36 |  |  |  |  | -0.43 | 0.54 | 0.41 |  |  |  |  |  |  |  |  | 0.50 |  |
| *f_Campylobacteraceae, g_Campylobacter* | -0.36 |  | 0.37 |  |  |  |  |  | 0.43 | -0.38 |  | 0.56 |  | 0.48 | 0.42 |  |  |  |  |  |  |
| *f_Comamonadaceae, g_Comamonas* |  |  |  |  |  |  |  |  |  |  |  |  |  |  |  |  |  |  |  |  | 0.35 |
| *f_Neisseriaceae* |  |  | -0.31 |  |  |  |  | 0.34 |  |  |  | -0.49 |  | -0.36 |  |  |  |  |  |  | -0.54 |
| *f_Pasteurellaceae* | -0.53 | -0.38 |  | -0.43 |  |  |  |  | 0.41 | -0.37 | -0.39 |  |  |  |  |  |  |  |  | -0.34 | -0.33 |
| *f_Pasteurellaceae, g_Bibersteinia* |  | -0.40 |  | -0.38 |  |  |  |  | 0.45 | -0.44 | -0.32 |  | -0.33 |  |  |  |  |  |  | -0.43 |  |
| *f_Rhodocyclaceae* |  |  | 0.33 |  |  |  |  |  |  |  |  |  |  | 0.37 | 0.43 |  |  |  |  |  |  |
| *f_Rhodospirillaceae* |  |  | 0.31 |  |  |  |  |  |  |  |  | 0.32 |  |  | 0.37 |  |  |  |  |  |  |
| *f_Succinivibrionaceae* | 0.52 | 0.35 |  | 0.38 |  |  |  |  | -0.48 | 0.56 | 0.45 |  |  |  |  |  |  |  |  | 0.53 |  |
| *g_Anaerobiospirillum* |  |  |  |  |  |  |  |  |  |  |  |  |  |  | 0.44 |  |  |  |  |  |  |
| *g_Ruminobacter* |  |  |  |  |  |  |  |  |  |  |  |  | 0.34 |  | 0.35 |  |  |  |  |  |  |
| *g_Succinimonas* |  |  |  |  |  |  |  |  |  |  | 0.31 |  |  |  | 0.47 |  |  |  |  |  |  |
| *g_Succinivibrio* | 0.49 | 0.37 |  | 0.33 |  |  |  |  | -0.49 | 0.39 | 0.45 |  |  |  |  |  | 0.38 |  |  | 0.43 |  |
| *p_Spirochaetae, f_Spirochaetaceae* |  | 0.39 |  | 0.34 |  |  |  |  |  |  |  |  |  |  |  |  |  |  |  |  |  |
| *g_Sphaerochaeta* |  |  |  |  |  |  | -0.35 |  |  |  | -0.33 |  |  |  |  |  |  |  |  |  |  |
| *g_Treponema* | 0.33 | 0.43 |  | 0.39 |  |  |  |  | -0.40 | 0.46 | 0.33 |  |  |  |  |  |  |  |  |  |  |
| *p_Synergistetes, f_Synergistaceae* |  |  |  |  |  | 0.33 |  |  |  | -0.33 |  |  |  |  |  |  |  |  |  |  |  |
| *g_Fretibacterium* |  | 0.42 | 0.55 | 0.45 |  |  |  | -0.37 |  |  |  | 0.35 | 0.36 | 0.62 | 0.66 |  |  | 0.32 |  |  | 0.33 |
| *g_Pyramidobacter* |  | -0.37 | -0.45 | -0.48 |  |  |  | 0.36 |  |  |  |  |  | -0.34 | -0.46 |  |  | -0.37 |  |  |  |
| *p_Tenericutes* |  |  | 0.59 |  |  |  |  |  |  | -0.36 |  | 0.31 | 0.34 | 0.67 | 0.69 |  |  |  |  |  |  |
| *f_Mollicutes_RF9_uncultured* |  |  |  |  |  |  |  |  |  |  |  |  |  |  |  | 0.43 |  |  | 0.40 |  |  |
| *f_NB1-n_uncultured* |  |  |  |  |  |  |  |  |  |  |  |  |  |  |  |  |  |  |  |  |  |
| *p_Lentisphaerae, f_Oligosphaeraceae* |  |  |  |  | 0.37 |  |  |  |  |  |  |  |  |  |  | 0.40 |  |  | 0.48 |  |  |
| p_Minor phyla | -0.46 |  | 0.49 |  |  |  |  |  | 0.46 | -0.54 |  | 0.46 |  | 0.56 | 0.46 |  |  |  |  |  |  |

^1^Parameters: pH, ammonia-N (mg/dl), total volatile fatty acids (mM), acetate (%), propionate (%), butyrate (%), lactate (mM) and average daily gain (kg/d) during the post-weaning (ADG-pw, weeks 6–13) and ﬁnishing periods (ADG-f, weeks 13–23). Microbial data were log10 transformed and Spearman’s correlations coefﬁcients ≥ 0.4 (green) or ≤ −0.4 (red) and P<0.001 were shown (n=72).

**Supplementary Table S4.** Effect of early-in-life rumen microbial inoculation and age on the rumen methanogens concentration, diversity and taxonomy.

| **Time** | **5 weeks** | | | | **7 weeks** | | | | **9 weeks** | | | |  | ***P*-value** | | |
| --- | --- | --- | --- | --- | --- | --- | --- | --- | --- | --- | --- | --- | --- | --- | --- | --- |
| **Inoculation^1^** | **CTL** | **AUT** | **RFF** | **RFC** | **CTL** | **AUT** | **RFF** | **RFC** | **CTL** | **AUT** | **RFF** | **RFC** | **s.e.d.** | **Inoc.** | **Time** | **IxT** |
| Diversity |  |  |  |  |  |  |  |  |  |  |  |  |  |  |  |  |
| Concentration (log10 copies/mg DM) | 5.15^e^ | 7.35^ab^ | 7.56^a^ | 6.29^c^ | 6.24^c^ | 7.18^ab^ | 7.08^b^ | 6.32^c^ | 5.18^e^ | 5.96^cd^ | 6.1^c^ | 5.73^de^ | 0.292 | <0.001 | <0.001 | <0.001 |
| Richness | 7.25^d^ | 11.4^c^ | 12^c^ | 13.6^bc^ | 6.63^d^ | 16.3^ab^ | 16.1^ab^ | 13.9^bc^ | 6.83^d^ | 17.6^a^ | 15.3^ab^ | 13.8^bc^ | 1.856 | <0.001 | 0.012 | 0.094 |
| Shannon index | 0.71 | 1.6 | 1.56 | 1.57 | 0.91 | 1.88 | 1.87 | 1.56 | 0.81 | 1.94 | 1.87 | 1.88 | 0.216 | <0.001 | 0.023 | 0.839 |
| Simpson index | 0.33 | 0.69 | 0.72 | 0.7 | 0.51 | 0.8 | 0.79 | 0.68 | 0.42 | 0.77 | 0.76 | 0.79 | 0.085 | <0.001 | 0.062 | 0.633 |
| Evenness | 0.14^c^ | 0.28^ab^ | 0.3^ab^ | 0.27^ab^ | 0.29^ab^ | 0.29^ab^ | 0.29^ab^ | 0.26^ab^ | 0.23^b^ | 0.27^ab^ | 0.28^ab^ | 0.31^a^ | 0.037 | 0.042 | 0.105 | 0.014 |
| Chao Index | 10.7 | 12.2 | 15.9 | 15.8 | 10.7 | 23 | 24.2 | 16.7 | 7.5 | 20.7 | 17.4 | 15.6 | 4.385 | 0.03 | 0.036 | 0.26 |
| Good´s coverage | 0.66 | 0.78 | 0.75 | 0.78 | 0.67 | 0.7 | 0.77 | 0.77 | 0.83 | 0.8 | 0.81 | 0.82 | 0.083 | 0.518 | 0.097 | 0.862 |
| Abundance (log10 sequences) |  |  |  |  |  |  |  |  |  |  |  |  |  |  |  |  |
| *f_Methanomassiliicoccaceae* | 1.48^cd^ | 2.43^a^ | 2.42^a^ | 2.38^ab^ | 2.41^a^ | 2.35^ab^ | 2.36^ab^ | 2.24^abc^ | 1.34^bcd^ | 2.22^abcd^ | 2.22^abcd^ | 1.48^d^ | 0.236 | 0.007 | <0.001 | 0.001 |
| *s_Methanomethylophilus_alvus* | 0^c^ | 0^c^ | 0^c^ | 0.31^bc^ | 0.06^c^ | 0.07^c^ | 0^c^ | 0^c^ | 1.09^ab^ | 0.65^abc^ | 1.21^a^ | 0^c^ | 0.224 | 0.049 | <0.001 | <0.001 |
| *s_Group10_sp* | 0^a^ | 1.55^c^ | 0.25^bc^ | 0.14^c^ | 0^ab^ | 0.98^c^ | 0.46^bc^ | 0.21^bc^ | 0^bc^ | 0.59^c^ | 0.67^abc^ | 0^c^ | 0.238 | <0.001 | 0.383 | 0.005 |
| *s_Group11_sp* | 0 | 0.15 | 0 | 0 | 0.13 | 0.29 | 0 | 0 | 0.48 | 0.04 | 0 | 0 | 0.127 | 0.109 | 0.316 | 0.009 |
| *s_ Group12_sp* | 0^b^ | 0^b^ | 0.08^ab^ | 0.45^a^ | 0^b^ | 0.50^a^ | 0.66^a^ | 0.19^ab^ | 0^b^ | 1.10^a^ | 0.15^ab^ | 0^b^ | 0.219 | 0.009 | 0.094 | <0.001 |
| *s_Group8_sp* | 1.48^c^ | 0.63^a^ | 0^bc^ | 0^c^ | 1.33^c^ | 0.81^ab^ | 0.3^bc^ | 0^bc^ | 0.32^c^ | 1.24^bc^ | 1.06^abc^ | 0.81^c^ | 0.329 | 0.003 | 0.026 | <0.001 |
| *s_Group9_sp* | 0^c^ | 1.37^a^ | 2.17^a^ | 2.1^a^ | 1.56^a^ | 1.97^a^ | 2.19^a^ | 2.09^a^ | 0.36^bc^ | 1.97^a^ | 1.66^a^ | 1.24^ab^ | 0.301 | <0.001 | <0.001 | <0.001 |
| *s_Methanomassiliicoccaceae spp.* | 0.04^d^ | 1.98^a^ | 1.91^ab^ | 1.5^ab^ | 0.54^cd^ | 1.62^ab^ | 1.51^ab^ | 1.47^ab^ | 0.05^d^ | 1.19^abc^ | 1.03^bc^ | 0.04^d^ | 0.267 | <0.001 | <0.001 | 0.006 |
| *f_Methanobacteriaceae* | 2.34^ab^ | 2.34^ab^ | 2.27^ab^ | 2.17^b^ | 2.23^ab^ | 2.41^ab^ | 2.38^ab^ | 2.38^ab^ | 2.57^ab^ | 2.31^ab^ | 2.5^ab^ | 2.66^a^ | 0.118 | 0.955 | 0.001 | 0.037 |
| *g_Methanobrevibacter* | 2.34^ab^ | 2.32^ab^ | 1.9^b^ | 2.06^ab^ | 2.23^ab^ | 2.31^ab^ | 1.98^ab^ | 2.04^ab^ | 2.42^ab^ | 2.11^ab^ | 2.42^ab^ | 2.55^a^ | 0.159 | 0.053 | 0.009 | 0.017 |
| *s_Methanobrevibacter_bovis_koreani* | 0^b^ | 0^b^ | 0^b^ | 0^b^ | 0^b^ | 0^b^ | 0^b^ | 0^b^ | 0^b^ | 0.11^b^ | 0.76^a^ | 1.03^a^ | 0.137 | <0.001 | <0.001 | <0.001 |
| *s_Methanobrevibacter_gottschalkii* | 2.34^a^ | 2.24^a^ | 1.82^c^ | 2.02^abc^ | 2.23^ab^ | 2.29^a^ | 1.73^c^ | 1.81^c^ | 2.41^a^ | 1.83^c^ | 1.92^bc^ | 2.37^a^ | 0.191 | <0.001 | 0.447 | 0.039 |
| *s_Methanobrevibacter_oralis* | 0 | 0 | 0 | 0 | 0 | 0 | 0 | 0 | 0 | 0 | 0 | 0.09 | 0.032 | 0.416 | 0.31 | 0.358 |
| *s_Methanobrevibacter_ruminantium* | 0 | 0.84 | 0.85 | 0.8 | 0.04 | 0.32 | 1.25 | 1.12 | 0.36 | 1.7 | 1.93 | 1.77 | 0.343 | <0.001 | <0.001 | 0.27 |
| *s_Methanobrevibacter spp.* | 0^b^ | 0^b^ | 0^b^ | 0.45^b^ | 0^b^ | 0.19^b^ | 0^b^ | 0.62^b^ | 0^b^ | 0^b^ | 0^b^ | 1.43^a^ | 0.203 | <0.001 | 0.005 | <0.001 |
| *g_Methanosphaera* | 0.1 | 0.31 | 0 | 0.22 | 0 | 0.24 | 0.48 | 0.76 | 1.72 | 1.42 | 1.36 | 1.33 | 0.290 | 0.69 | <0.001 | 0.139 |
| *s_Methanosphaera_sp_ISO3-F5* | 0.1 | 0.26 | 0 | 0.22 | 0 | 0.12 | 0.48 | 0.76 | 1.58 | 1.31 | 1.28 | 1.33 | 0.291 | 0.534 | <0.001 | 0.187 |
| *s_Methanosphaera_stadtmanae* | 0^b^ | 0.05^b^ | 0^b^ | 0^b^ | 0^b^ | 0.12^b^ | 0^b^ | 0^b^ | 0.2^b^ | 0.78^a^ | 0.75^a^ | 0^b^ | 0.158 | 0.003 | <0.001 | 0.007 |
| *s_Methanomicrobium_mobile* | 0^b^ | 0.47^ab^ | 1.45^a^ | 0.8^ab^ | 0^b^ | 1.30^a^ | 1.48^a^ | 1.35^a^ | 0^b^ | 1.06^ab^ | 0.76^ab^ | 0^b^ | 0.317 | <0.001 | 0.001 | 0.006 |
| *f_Methanosarcinaceae* | 0^c^ | 0^c^ | 0.38^abc^ | 0.32^bc^ | 0^c^ | 0^c^ | 0.59^ab^ | 0.91^a^ | 0^c^ | 0.42^abc^ | 0^c^ | 0^c^ | 0.165 | 0.002 | 0.002 | <0.001 |
| *s_Methanimicrococcus_blatticola* | 0^c^ | 0^c^ | 0.38^abc^ | 0.32^bc^ | 0^c^ | 0^c^ | 0.59^ab^ | 0.91^a^ | 0^c^ | 0.42^abc^ | 0^c^ | 0^c^ | 0.165 | 0.002 | 0.002 | <0.001 |

^1^Goat kids inoculated (I) with fresh rumen fluid from adult goats adapted to forage-rich (RFF) or concentrate-rich diets (RFC), autoclaved rumen fluid (AUT) or absence of inoculation as control (CTL) and sampled at 5, 7 and 9 weeks of age.^a-f^ Means within a row with different superscript differ (*P*<0.05) based on the Bonferroni test.

**Supplementary Table S5.** Effect of early-in-life rumen microbial inoculation and age on the rumen protozoal concentration, diversity and taxonomy.

| **Time** | **5 weeks** | | | | **7 weeks** | | | | **9 weeks** | | | |  | ***P-value*** | | |
| --- | --- | --- | --- | --- | --- | --- | --- | --- | --- | --- | --- | --- | --- | --- | --- | --- |
| **Inoculation^1^** | **CTL** | **AUT** | **RFF** | **RFC** | **CTL** | **AUT** | **RFF** | **RFC** | **CTL** | **AUT** | **RFF** | **RFC** | **s.e.d.** | **Inoc.** | **Time** | **AxT** |
| Diversity |  |  |  |  |  |  |  |  |  |  |  |  |  |  |  |  |
| Concentation (log10 copies/mg DM) | 0^e^ | 6.07^d^ | 8.05^ab^ | 8.00^ab^ | 0^e^ | 7.07^c^ | 8.15^ab^ | 8.33^a^ | 0^e^ | 6.91^c^ | 7.53^bc^ | 8.40^a^ | 0.3387 | <0.000 | 0.076 | 0.062 |
| Richness | 0 | 16.1 | 26.1 | 24.6 | 0 | 22.3 | 27.5 | 26.8 | 0 | 22.0 | 22.3 | 26.4 | 2.821 | <0.001 | 0.194 | 0.180 |
| Shannon index | 0^d^ | 1.17^c^ | 2.17^ab^ | 2.10^ab^ | 0^d^ | 1.77^ab^ | 2.22^a^ | 2.16^ab^ | 0^d^ | 1.70^b^ | 1.93^ab^ | 2.16^ab^ | 0.230 | <0.001 | 0.185 | 0.050 |
| Simpson index | 0^d^ | 0.52^c^ | 0.84^a^ | 0.82^a^ | 0^d^ | 0.75^a^ | 0.84^a^ | 0.82^a^ | 0^d^ | 0.63^b^ | 0.75^ab^ | 0.81^a^ | 0.071 | <0.001 | 0.124 | 0.029 |
| Evenness | 0 | 0.42 | 0.67 | 0.66 | 0 | 0.57 | 0.67 | 0.66 | 0 | 0.52 | 0.63 | 0.66 | 0.060 | <0.001 | 0.311 | 0.185 |
| Chao Index | 0 | 16.9 | 28.8 | 25.3 | 0 | 25.5 | 31.7 | 32.9 | 0 | 23.7 | 23.9 | 27.4 | 3.203 | <0.001 | 0.012 | 0.133 |
| Good´s coverage | 0 | 0.89 | 0.86 | 0.92 | 0 | 0.85 | 0.87 | 0.83 | 0 | 0.86 | 0.87 | 0.90 | 0.034 | <0.001 | 0.128 | 0.252 |
| Abundance (log 10 sequences) |  |  |  |  |  |  |  |  |  |  |  |  |  |  |  |  |
| *f_Ophryoscolecidae* | 0^c^ | 3.73^a^ | 3.69^a^ | 3.67^ab^ | 0^c^ | 3.7^a^ | 3.64^ab^ | 3.67^a^ | 0^c^ | 3.68^a^ | 3.54^b^ | 3.65^ab^ | 0.038 | <0.001 | 0.054 | 0.042 |
| *g_Entodinium* | 0 | 3.64 | 3.50 | 3.41 | 0 | 3.53 | 3.28 | 3.36 | 0 | 3.50 | 3.19 | 3.35 | 0.090 | <0.001 | 0.033 | 0.286 |
| *g_Ophryoscolex* | 0 | 1.93 | 2.43 | 2.82 | 0 | 2.08 | 3.14 | 3.20 | 0 | 2.13 | 2.71 | 3.18 | 0.393 | <0.001 | 0.229 | 0.892 |
| *g_Diplodinium* | 0^c^ | 0.22^bc^ | 1.39^ab^ | 0.70^abc^ | 0^c^ | 0.90^abc^ | 1.47^a^ | 1.05^abc^ | 0^c^ | 0.83^abc^ | 0.53^abc^ | 0^c^ | 0.367 | <0.001 | 0.021 | 0.048 |
| *g_Polyplastron* | 0 | 1.98 | 2.55 | 2.37 | 0 | 2.26 | 2.11 | 2.47 | 0 | 1.90 | 1.64 | 2.27 | 0.351 | <0.001 | 0.165 | 0.196 |
| *g_Enoploplastron* | 0^c^ | 1.32^abc^ | 2.40^a^ | 1.78^ab^ | 0^c^ | 1.21^abc^ | 2.00^ab^ | 1.71^ab^ | 0^c^ | 1.46^abc^ | 0.94^bc^ | 0.70^bc^ | 0.422 | <0.001 | <0.001 | 0.003 |
| *f_Buetschliidae* | 0^d^ | 0.44^cd^ | 2.24^ab^ | 2.28^ab^ | 0^d^ | 1.23^bc^ | 2.82^a^ | 2.6^a^ | 0^d^ | 1.95^ab^ | 2.99^a^ | 2.88^a^ | 0.331 | <0.001 | <0.001 | 0.037 |
| *g_Isotricha* | 0^c^ | 0.19^c^ | 1.65^ab^ | 1.67^ab^ | 0^c^ | 1.01^bc^ | 2.29^a^ | 1.98^ab^ | 0^c^ | 1.73^ab^ | 2.71^a^ | 2.53^a^ | 0.338 | <0.001 | <0.001 | 0.045 |
| *g_Dasytricha* | 0 | 0.24 | 1.92 | 1.69 | 0 | 1.12 | 2.63 | 2.17 | 0 | 1.67 | 2.05 | 2.59 | 0.403 | <0.001 | 0.003 | 0.057 |
| *g_uncultured* | 0 | 1.27 | 1.96 | 2.31 | 0 | 2.01 | 2.14 | 2.11 | 0 | 2.03 | 1.74 | 2.06 | 0.378 | <0.001 | 0.635 | 0.326 |

^1^Goat kids inoculated (I) with fresh rumen fluid from adult goats adapted to forage-rich (RFF) or concentrate-rich diets (RFC), autoclaved rumen fluid (AUT) or absence of inoculation as control (CTL) and sampled at 5, 7 and 9 weeks of age.^a-f^ Means within a row with different superscript differ (*P*<0.05) based on the Bonferroni test.

**Supplementary Table S6.** Effect of early-in-life rumen microbial inoculation and age on the rumen anaerobic fungal concentration, diversity and taxonomy.

| **Time** | **5 weeks** | | | | **7 weeks** | | | | **9 weeks** | | | |  | ***P*-value** | | |
| --- | --- | --- | --- | --- | --- | --- | --- | --- | --- | --- | --- | --- | --- | --- | --- | --- |
| **Inoculation^1^** | **CTL** | **AUT** | **RFF** | **RFC** | **CTL** | **AUT** | **RFF** | **RFC** | **CTL** | **AUT** | **RFF** | **RFC** | **SED1** | **Inoc.** | **Time** | **AxT** |
| Diversity |  |  |  |  |  |  |  |  |  |  |  |  |  |  |  |  |
| Concentation (log10 copies/mg DM) | 5.77^cde^ | 5.35^ef^ | 6.28^ab^ | 6.09^abc^ | 5.96^bc^ | 5.77^cd^ | 6.40^a^ | 5.93^bc^ | 5.46^def^ | 5.37^ef^ | 5.42^def^ | 5.24^f^ | 0.203 | 0.006 | <0.001 | 0.006 |
| Richness | 16.5 | 14.8 | 12.4 | 13.2 | 14.9 | 13.8 | 12.7 | 14.4 | n.m. | n.m. | n.m. | n.m. | 3.885 | 0.765 | 0.989 | 0.942 |
| Shannon index | 1.06 | 0.89 | 1.01 | 1.38 | 1.17 | 1.18 | 1.08 | 1.35 | n.m. | n.m. | n.m. | n.m. | 0.305 | 0.554 | 0.395 | 0.889 |
| Simpson index | 0.46 | 0.40 | 0.47 | 0.61 | 0.56 | 0.53 | 0.48 | 0.64 | n.m. | n.m. | n.m. | n.m. | 0.126 | 0.515 | 0.159 | 0.742 |
| Evenness | 0.38 | 0.31 | 0.40 | 0.54 | 0.44 | 0.44 | 0.43 | 0.51 | n.m. | n.m. | n.m. | n.m. | 0.091 | 0.324 | 0.194 | 0.554 |
| Chao Index | 20.5 | 34.4 | 17.3 | 14.5 | 18.0 | 19.7 | 18.7 | 18.8 | n.m. | n.m. | n.m. | n.m. | 7.753 | 0.523 | 0.686 | 0.308 |
| Good´s coverage | 0.74 | 0.61 | 0.71 | 0.84 | 0.74 | 0.76 | 0.67 | 0.71 | n.m. | n.m. | n.m. | n.m. | 0.090 | 0.672 | 0.718 | 0.085 |
| Abundance (log10 sequences) |  |  |  |  |  |  |  |  |  |  |  |  |  |  |  |  |
| *g_Caecomyces* | 0.93 | 1.49 | 0 | 0.28 | 0.70 | 1.03 | 0.04 | 0 | n.m. | n.m. | n.m. | n.m. | 0.503 | 0.062 | 0.247 | 0.433 |
| *g_Capnodiales* | 0 | 0.64 | 0 | 0.38 | 0 | 0 | 0 | 0 | n.m. | n.m. | n.m. | n.m. | 0.165 | 0.325 | 0.113 | 0.237 |
| *g_Neocallimastix* | 0.81^a^ | 0.82^a^ | 0.04^b^ | 0^b^ | 0.65^a^ | 0.20^ab^ | 0.04^b^ | 0.04^b^ | n.m. | n.m. | n.m. | n.m. | 0.342 | 0.187 | 0.247 | 0.037 |
| *g_Orpinomyces* | 0 | 0 | 0.42 | 0 | 0 | 0.90 | 1.39 | 0.85 | n.m. | n.m. | n.m. | n.m. | 0.292 | 0.030 | 0.023 | 0.496 |
| *g_Piromyces* | 0 | 0.60 | 1.07 | 1.58 | 0 | 1.62 | 0.57 | 2.00 | n.m. | n.m. | n.m. | n.m. | 0.537 | <0.001 | 0.524 | 0.303 |
| *g_Neocallimastigaceae spp.* | 2.70 | 1.88 | 2.76 | 2.79 | 2.90 | 1.37 | 2.87 | 2.09 | n.m. | n.m. | n.m. | n.m. | 0.362 | 0.007 | 0.195 | 0.388 |

^1^Goat kids inoculated (I) with fresh rumen fluid from adult goats adapted to forage-rich (RFF) or concentrate-rich diets (RFC), autoclaved rumen fluid (AUT) or absence of inoculation as control (CTL) and sampled at 5, 7 and 9 weeks of age.^a-f^ Means within a row with different superscript differ (*P*<0.05) based on the Bonferroni test; n.m., not measured.

**Supplementary Table S7.** Descriptive statistics of the metadata used in the distance-based redundancy analyses and spearman correlations with microbial taxa abundance.

|  |  | **5 weeks** | | | | **7 weeks** | | | | **9 weeks** | | | |
| --- | --- | --- | --- | --- | --- | --- | --- | --- | --- | --- | --- | --- | --- |
| **Abreviation^1^** | **Units** | **Min** | **Max** | **Mean** | **SD** | **Min** | **Max** | **Mean** | **SD** | **Min** | **Max** | **Mean** | **SD** |
| ADG | kg/d | 0.04 | 0.22 | 0.16 | 0.05 | 0.08 | 0.25 | 0.19 | 0.04 | 0.08 | 0.29 | 0.19 | 0.05 |
| ADG-f | kg/d | 0.12 | 0.29 | 0.20 | 0.04 | 0.00 | 0.26 | 0.14 | 0.06 | 0.11 | 0.37 | 0.23 | 0.06 |
| FE | kg/Mcal ME | 0.05 | 0.29 | 0.19 | 0.06 | 0.08 | 0.26 | 0.18 | 0.04 | 0.10 | 0.34 | 0.21 | 0.06 |
| FE-f | kg/Mcal ME | 0.14 | 0.32 | 0.22 | 0.05 | 0.01 | 0.62 | 0.37 | 0.15 | 0.09 | 0.33 | 0.18 | 0.05 |
| Milk | L/d | 1.09 | 1.58 | 1.38 | 0.13 | 1.49 | 1.88 | 1.65 | 0.11 | 0.00 | 0.00 | 0.00 | 0.00 |
| Concentrate | g/d | 3.34 | 24.18 | 9.75 | 5.64 | 8.14 | 62.22 | 25.71 | 13.96 | 117.9 | 274.2 | 171.2 | 44.67 |
| Concentrate-f | g/d | 8.14 | 62.22 | 25.71 | 13.96 | 117.9 | 274.2 | 171.2 | 44.67 | 299.3 | 488.9 | 373.6 | 54.40 |
| Forage | g/d | 4.68 | 20.16 | 11.56 | 4.82 | 9.75 | 23.61 | 16.19 | 4.01 | 68.62 | 139.99 | 98.45 | 20.25 |
| Forage-f | g/d | 9.75 | 23.61 | 16.19 | 4.01 | 68.62 | 140.0 | 98.45 | 20.25 | 143.4 | 285.5 | 209.4 | 36.99 |
| DMI | g/d | 6.94 | 33.58 | 15.53 | 7.34 | 22.54 | 75.90 | 41.91 | 14.50 | 202.4 | 344.8 | 269.7 | 45.53 |
| DMI-f | g/d | 22.54 | 75.90 | 41.91 | 14.50 | 202.4 | 344.8 | 269.7 | 45.53 | 493.5 | 714.8 | 583.0 | 71.90 |
| pH | pH units | 5.76 | 7.13 | 6.71 | 0.24 | 5.48 | 6.92 | 6.57 | 0.29 | 5.79 | 7.40 | 6.68 | 0.45 |
| Ammonia-N | mg/dL | 11.82 | 49.71 | 27.49 | 8.65 | 12.22 | 50.48 | 29.18 | 9.49 | 0.03 | 24.15 | 8.80 | 6.65 |
| VFA | mM | 10.58 | 76.72 | 29.45 | 15.01 | 12.58 | 52.01 | 30.81 | 13.09 | 17.79 | 79.03 | 51.28 | 14.90 |
| Acetate | % | 54.30 | 81.30 | 73.87 | 5.75 | 58.80 | 84.90 | 69.58 | 6.81 | 42.80 | 67.30 | 56.93 | 6.58 |
| Propionate | % | 9.40 | 32.70 | 14.43 | 3.86 | 9.80 | 25.90 | 15.81 | 3.61 | 16.30 | 39.70 | 24.94 | 6.47 |
| Butyrate | % | 0.70 | 18.70 | 4.87 | 4.11 | 1.00 | 18.00 | 8.67 | 4.62 | 5.90 | 23.10 | 13.80 | 4.65 |
| OBCVFA | % | 3.80 | 14.00 | 6.83 | 1.93 | 3.90 | 11.00 | 5.94 | 1.69 | 2.20 | 7.30 | 4.29 | 1.12 |
| Bacteria | log copies/mg DM | 9.92 | 12.03 | 10.66 | 0.35 | 8.97 | 11.32 | 10.47 | 0.45 | 9.64 | 11.03 | 10.47 | 0.35 |
| Bacterial-R | OTUs | 111.0 | 514.0 | 300.8 | 130.6 | 183.0 | 653.0 | 389.1 | 154.9 | 97.0 | 561.0 | 285.7 | 130.2 |
| Methanogens | log copies/mg DM | 2.64 | 9.02 | 6.46 | 1.77 | 2.83 | 8.47 | 6.59 | 1.37 | 2.38 | 7.73 | 5.84 | 1.20 |
| Methanogens-R | OTUs | 1.00 | 18.00 | 11.06 | 4.76 | 4.00 | 22.00 | 13.21 | 5.20 | 4.00 | 21.00 | 13.36 | 4.75 |
| Protozoa | log copies/mg DM | 2.98 | 8.85 | 6.63 | 1.99 | 3.46 | 9.27 | 6.92 | 1.87 | 2.96 | 9.19 | 6.84 | 2.04 |
| Protozoal-R | OTUs | 0.00 | 31.00 | 16.72 | 11.05 | 0.00 | 34.00 | 19.13 | 12.46 | 0.00 | 35.00 | 17.66 | 12.76 |
| Fungi | log copies/mg DM | 3.48 | 7.00 | 5.89 | 0.81 | 3.48 | 7.39 | 6.25 | 0.67 | 3.40 | 7.25 | 5.42 | 0.98 |
| Fungal-R | OTUs | 3.00 | 30.00 | 14.20 | 6.26 | 5.00 | 34.00 | 13.92 | 7.63 |  |  |  |  |
| BHB | mM | 0.95 | 5.25 | 2.57 | 1.13 | 1.31 | 4.98 | 3.07 | 0.76 | 1.31 | 3.95 | 2.49 | 0.73 |
| Glucose | mg/dL | 44.65 | 130.1 | 95.83 | 20.75 | 70.79 | 104.32 | 90.29 | 8.71 | 76.04 | 99.17 | 87.84 | 5.95 |
| DOMI | g/d |  |  |  |  |  |  |  |  | 342.58 | 498.73 | 435.04 | 45.00 |
| DNI | g/d |  |  |  |  |  |  |  |  | 14.22 | 20.42 | 17.42 | 1.94 |
| DHCI | g/d |  |  |  |  |  |  |  |  | 43.71 | 70.31 | 58.07 | 6.84 |
| DCI | g/d |  |  |  |  |  |  |  |  | 25.33 | 61.12 | 41.50 | 10.34 |

^1^ADG, average daily gain; -f, following week; FE, feed efficiency; Concentrate, concentrate-feed intake; Forage, forage-feed intake; DMI, dry mater intake; NH_3_, rumen ammonia; VFA, volatile fatty acids; OBCVFA, odd and branched chain VFA; -R, richness; BHB, plasma β-hydroxybutyrate; Glucose, plasma glucose; DOMI, digestible organic matter intake; DNI; digestible nitrogen intake; DHCI, digestible hemicellulose intake; DCI, digestible cellulose intake.

**Supplementary Table S8.** Primers used for quantitative PCR and Next Generation Sequencing.

| Target | Ref. | Name Primer | Forward Primer | Name | Reverse Primer | Amplicon (bp) |
| --- | --- | --- | --- | --- | --- | --- |
| Quantitative PCR |  |  |  |  |  |  |
| Total bacteria | [1] | 1048F | GTGSTGCAYGGYTGTCGTCA | 1175R | ACGTCRTCCMCACCTTCCTC | 150 |
| Methanogens | [2] | qmcrA-F | TTCGGTGGATCDCARAGRGC | qmcrA-R | GBARGTCGWAWCCGTAGAATCC | 140 |
| Protozoa | [3] | P-SSU-316f | GCTTTCGWTGGTAGTGTATT | PS-SU-539r | CTTGCCCTCYAATCGTWCT | 223 |
| Anaerobic fungi | [4] | qPCR fungi-F | GAGGAAGTAAAAGTCGTAACAAGGTTTC | qPCR fungi-R | CAAATTCACAAAGGGTAGGATGATT | 120 |
| Sequencing |  |  |  |  |  |  |
| Bacteria | [5] | V3_F357 | CCTACGGGAGGCAGCAG | V5_926 | CCGTCAATTCMTTTRAGT | 570 |
| Methanogens | [6] | Arch349F | GYGCASCAGKCGMGAAW | Arch806R | GGACTACVSGGGTATCTAAT | 457 |
| Protozoa | [7] | F566Euk | CAGCAGCCGCGGTAATTCC | R1200Euk | CCCGTGTTGAGTCAAATTAAGC | 660+var. |
| Anaerobic fungi | [8] | ITS3 | GCATCGATGAAGAACGCAGC | ITS4 | TCCTCCGCTTATTGATATGC | 356+var. |

**References**

1. Maeda H, Fujimoto C, Haruki Y, et al. Quantitative real-time PCR using TaqMan and SYBR Green for *Actinobacillus actinomycetemcomitans , Porphyromonas gingivalis , Prevotella intermedia* , tetQ gene and total bacteria. *FEMS Immunol Med Microbiol* 2003;39:81–86.
2. Denman, S.E., N. Tomkins, C.S. McSweeney, *Quantitation and diversity analysis of ruminal methanogenic populations in response to the antimethanogenic compound bromochloromethane.* Fems Microbiol Ecol 2007;**62**:313-22.

3. Sylvester JT, Karnati SKR, Yu ZT et al. Development of an assay to quantify rumen ciliate protozoal biomass in cows using real-time PCR. *J Nutr* 2004;**134**: 3378-84.

4. Denman SE, McSweeney CS. Development of a real-time PCR assay for monitoring anaerobic fungal and cellulolytic bacterial populations within the rumen. *FEMS Microbiol Ecol* 2006;**58**: 572-82.

5. Sim K, Cox MJ, Wopereis H, et al. Improved detection of bifidobacteria with optimised 16S rRNA-gene based pyrosequencing. *PloS one* 2012;**7**:e32543.

6. Gantner S, Andersson AF, Alonso-Sáez L, et al. Novel primers for 16S rRNA-based archaeal community analyses in environmental samples. *J Microbiol Meth* 2011;**84**: 12-18.

7. Hadziavdic K, Lekang K, Lanzen A, et al. Characterization of the 18S rRNA gene for designing universal eukaryote specific primers. *PloS one* 2014;**9**: e87624.

8. De Beeck MO, Lievens B, Busschaert P, et al. Comparison and validation of some ITS primer pairs useful for fungal metabarcoding studies. *PloS one* 2014;**9**: e97629.


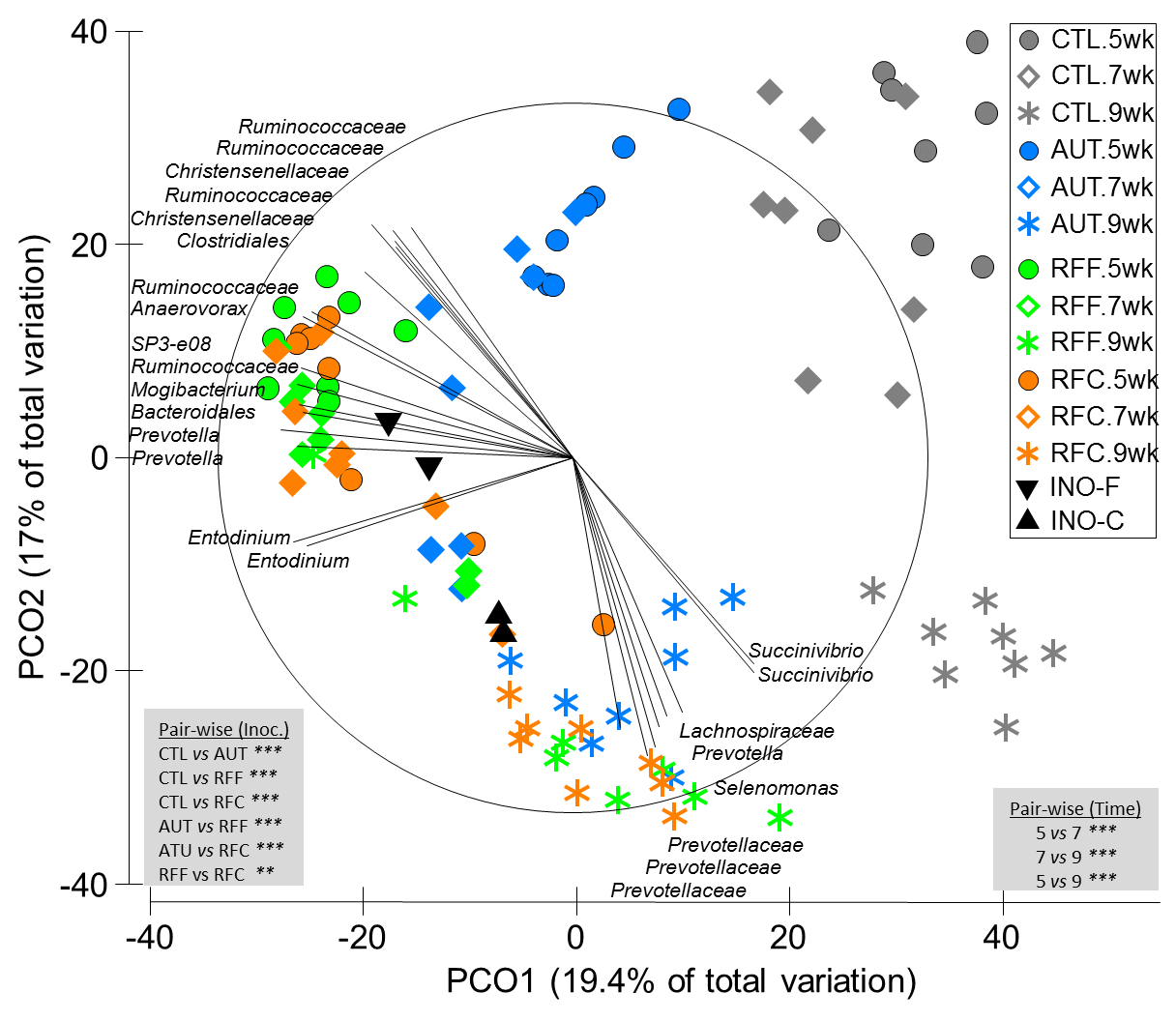


**Supplementary Figure S1**. Principal co-ordinates analysis illustrating the inoculation effects on the multi-kingdom rumen microbioma. Analysis included bacteria, methanogens, protozoa and anaerobic fungi data along with the most discriminant OTUs (*ρ* > 0.75). Pair-wise PERMANOVA values are provided in grey boxes based on the Bray-Curtis dissimilarity. ****P* < 0.001; ***P* < 0.01. Goat kids inoculated (I) with fresh rumen fluid from adult goats adapted to forage-rich (RFF) or concentrate-rich diets (RFC), autoclaved rumen fluid (AUT) or absence of inoculation as control (CTL) and sampled at 5, 7 and 9 weeks of age.

1. Maeda, H., et al., *Quantitative real-time PCR using TaqMan and SYBR Green for Actinobacillus actinomycetemcomitans , Porphyromonas gingivalis , Prevotella intermedia , tetQ gene and total bacteria.* FEMS Immunol Med Microbiol, 2003. **39**: p. 81 – 86.

2. Denman, S.E., N. Tomkins, and C.S. McSweeney, *Quantitation and diversity analysis of ruminal methanogenic populations in response to the antimethanogenic compound bromochloromethane.* Fems Microbiology Ecology, 2007. **62**: p. 313-322.

3. Sylvester, J.T., et al., *Development of an assay to quantify rumen ciliate protozoal biomass in cows using real-time PCR.* Journal of Nutrition, 2004. **134**(12): p. 3378-3384.

4. Denman, S.E. and C.S. McSweeney, *Development of a real-time PCR assay for monitoring anaerobic fungal and cellulolytic bacterial populations within the rumen.* FEMS Microbiology Ecology, 2006. **58**(3): p. 572-582.

5. Sim, K., et al., *Improved detection of bifidobacteria with optimised 16S rRNA-gene based pyrosequencing.* PloS one, 2012. **7**(3): p. e32543.

6. Gantner, S., et al., *Novel primers for 16S rRNA-based archaeal community analyses in environmental samples.* Journal of Microbiological Methods, 2011. **84**(1): p. 12-18.

7. Hadziavdic, K., et al., *Characterization of the 18S rRNA gene for designing universal eukaryote specific primers.* PloS one, 2014. **9**(2): p. e87624.

8. De Beeck, M.O., et al., *Comparison and validation of some ITS primer pairs useful for fungal metabarcoding studies.* PloS one, 2014. **9**(6): p. e97629.
